# Mesoporous Silica Nanoparticles as Carriers for Therapeutic Biomolecules

**DOI:** 10.3390/pharmaceutics12050432

**Published:** 2020-05-07

**Authors:** Rafael R. Castillo, Daniel Lozano, María Vallet-Regí

**Affiliations:** 1 Departamento de Química en Ciencias Farmacéuticas, Facultad de Farmacia, Universidad Complutense de Madrid, Plaza Ramón y Cajal s/n, 28040 Madrid, Spain; rafcas01@ucm.es (R.R.C.); danlozan@ucm.es (D.L.); 2 Centro de Investigación Biomédica en Red—CIBER, 28029 Madrid, Spain; 3 Instituto de Investigación Sanitaria Hospital 12 de Octubre—imas12, 28041 Madrid, Spain

**Keywords:** mesoporous silica, therapeutic biomolecules, proteins, peptides, nucleic acids, glycans

## Abstract

The enormous versatility of mesoporous silica nanoparticles permits the creation of a large number of nanotherapeutic systems for the treatment of cancer and many other pathologies. In addition to the controlled release of small drugs, these materials allow a broad number of molecules of a very different nature and sizes. In this review, we focus on biogenic species with therapeutic abilities (proteins, peptides, nucleic acids, and glycans), as well as how nanotechnology, in particular silica-based materials, can help in establishing new and more efficient routes for their administration. Indeed, since the applicability of those combinations of mesoporous silica with bio(macro)molecules goes beyond cancer treatment, we address a classification based on the type of therapeutic action. Likewise, as illustrative content, we highlight the most typical issues and problems found in the preparation of those hybrid nanotherapeutic materials.

## 1. Introduction

Since the very first example reported on nanometric mesoporous silica as a drug delivery material [1], many examples were subsequently reported including a broad number of chemical species. One of them includes biomacromolecules, which play a capital role in living beings, as they are responsible for biorecognition [2], signal transduction [3], and replication routes; hence, they are responsible for the adequate development of tissues and organs. Such is the importance of these species that even the immune system and hormone signaling are based on specific affinity interactions between biomacromolecules (BMs). From a therapeutic point of view, hijacking such ligands and receptors may be useful to regulate unbalanced systems, as well as develop new-generation therapeutic nanodevices in oncology [4,5] cancer immunotherapy [6,7,8], and gene therapy [9,10,11], among many others.

Unfortunately, most bioactive macromolecules are highly labile in vivo, as a self-regulation mechanism to avoid massive damages. Therefore, to use them, it is necessary to implement chemical modifications or to design vehicles capable of ensuring an adequate preservation and, hence, a long-lasting effect. Among known carriers, viruses have the best performance, although at the expense of having a great associated risk in handling and containment. Additionally, they are only suitable for nucleic acids (NAs), being useless for protein and peptide delivery. To overcome these limitations, nanoparticles emerged as promising vectors for nucleotides, as well as peptides and proteins. This is a consequence of two complementary aspects. On one hand, their size permits establishing intimate interactions with cell’s membranes and the receptors therein. On the other hand, they exhibit the possibility to establish non-conventional interactions between particles, cargoes, and target cells.

**Figure 1:**
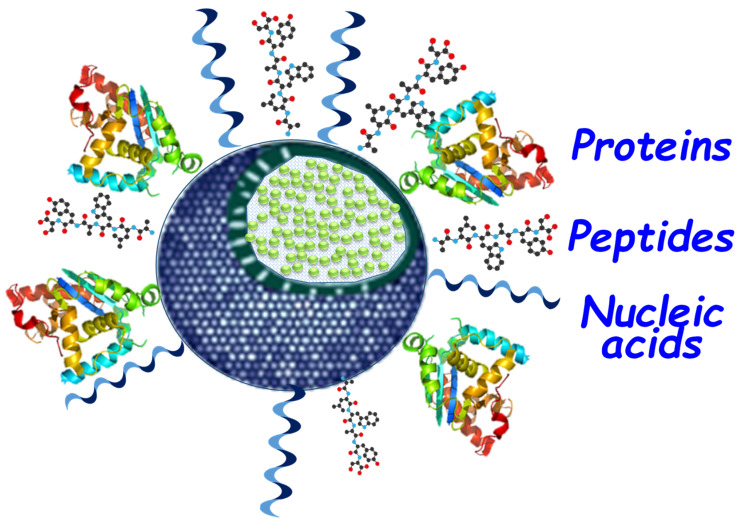
Main groups of therapeutic biomolecules that are possible to deliver using mesoporous nanosilica technology.

Amongst all known materials, mesoporous silica nanoparticles (MSNs) arose as promising drug delivery platforms because of their outstanding biocompatibility, their degradability, and their great chemical and biological robustness. Moreover, the unique porous structure of MSNs permits establishing host–guest interactions of high interest for drug delivery purposes, as they allow creating protective environments for labile molecules. In addition to those, the current silica-based nanotechnology also permits creating particles with variable diameters [12,13], pore sizes [14], and structures [15], which permit fine-tuning the final application of the nanosystems, especially those intended to deliver big cargoes such as those reviewed herein. Moreover, MSNs and related hybrid particles with SiO_2_ coatings also permit easily tuning the resulting outer layers of nanosystems to enhance biochemical stability and, hence, to reduce side effects and potential toxicities [16,17,18]. Particle diameter is one of the most critical parameters for achieving a successful therapy. Typically, the accepted window of diameters for cancer treatment comprises particles in a range between 50 and 300 nm, in which the enhanced permeation and retention effect operates. However, depending on the final purpose of the nanosystem, such values may be narrowed. For instance, in cases where a superior trafficking is desired, smaller particles would behave better, while, in nanosystems intended as biomolecule reservoirs, the diameter must unavoidably be increased. Regarding nanoparticle-based drug delivery, we recently reviewed how mesoporous silica-based nanosystems are suitable platforms to combine two or more chemical species, outranging the pharmacological profile of free species [19,20]. However, despite the possible therapeutic improvement, their efficiency and long-term stability could be compromised if the BMs are not properly protected or fully exposed to white blood cells and immune systems. The scarce protection provided by most solid nanoparticles highlights once again the importance of MSNs [21] as platforms for the development of non-viral vectors and protein carriers, whose particular porous morphology can provide a protective environment for those labile molecules, although the typical porosity (2–3 nm) of MSNs may be tuned in order to host the biggest molecules [22] (Figure 1).

As introduced, the use of MSNs in biomedicine has a huge potential impact; in addition to acting as carriers, they also permit creating fancy structures with most functional nanomaterials. However, despite this versatility, the permeation of these materials into clinical trials is still limited [23,24]; this is not caused by poor biocompatibility, but rather by the impossibility of establishing reliable comparisons between different systems, as accounted by Florence [25]. In fact, as can be observed from the growing number of in vivo experiments using MSNs carried out by many groups worldwide, it is logical to assume that they have adequate performance in living systems; thus, MSNs will hopefully soon reach clinical practice.

## 2. Strategies to Deliver Biomacromolecules with Silica Nanoparticles

Loading effectiveness, cellular internalization, targeting, and cargo delivery are critical issues when developing a nanosystem with maximal therapeutic potential. As we previously mentioned, MSNs are ideal nanocarriers related to the load and delivery of biomolecules due to their unique properties, including shape, size, and surface chemistry [26]. Pore, channel, and cavity sizes can be modified in MSNs to increase the loading of therapeutic molecules [27]. The MSN surface can be functionalized with polyethyleneimine and poly-l-lysine or modified with targeting peptides or antibodies to improve cargo loading, cellular uptake, and endosomal scape rates [26]. The chemical and physical properties of MSNs are critical when designing a nanocarrier with therapeutic efficacy in biomolecule loading and delivery. In the last few years, surface nanoscale topography gained special attention due to the possibility of controlling the interactions between molecules and cells, in the process of molecular loading and cell internalization [28,29].

The chemical functionalization of silica is easily achieved through condensation processes employing functionalized alkoxysilanes. For the functionalization of surfaces, this silanization must be carried out before template removal, while the modification of mesopores could be achieved either via condensation during the template-driven synthesis of MSNs or after surface modification and template removal. In this latter case, it is important to account that pore constriction may occur if the process is not properly controlled. For the preparation of hollow MSNs (HMSNs), the most employed method is the solid template etching of an internal core, typically from solid silica, onto which a mesoporous layer is created. Pore and surface modification can be achieved in a parallel manner as done with conventional MSNs, while, for the functionalization of the internal space, this must be done prior to the formation of the mesoporous layer. Onto this slightly modified MSN, it would be possible to create additional modifications by linking chemical species through conventional chemical reactions. With this strategy, it was possible to create a huge number of functional nanosystems with pore nanogates [30], sensitive bonds [31], recognition elements [5,19], and charged surfaces able to undergo electrostatic interactions.

One common strategy to combine BMs and NPs is surface functionalization; it can be achieved either via a chemical bond or via an electrostatic interaction between charged counterparts. On MSNs, surface grafting is usually accomplished via direct condensation of the remaining Si–OH groups with functional silane reagents; these are typically modified trialkoxysilanes with aliphatic chains bearing an additional functional group. For the bonding of peptides and proteins, the most common functional groups are amino and carboxylate for amide coupling and maleimides for thiol-mediated binding [32]. Additionally, to complement the direct coupling approach, there are also many different bifunctional linkers available which are able to accomplish this task [33]. With regard to this coupling strategy, it is also important to remark the importance of controlling the bonding, which, if produced at the active region of proteins, may lead to inactivation. This is of particular importance when preparing antibody-targeted nanosystems [34,35] and sensors. On this topic, Landry and coworkers studied how the chemical linkage affected the specificity of cluster of differentiation 4 (CD4)-bonded proteins onto MSN against its target, the gp120 glycoprotein [36]. The authors proved that a conventional, unspecific, direct thiol–maleimide linkage behaved worse than a specific linkage placed far away from the active site.

The other approach for surface functionalization is electrostatic deposition. In this case, both nanocarriers and BMs need to establish strong electrostatic interactions through differently charged functional groups. This is of particular importance for NAs, whose permanent negative charge permits strong interactions with positively charged surfaces and polymers such as polyethyleneimine or chitosan among many others. Insights into this strategy are available in References [20,37]. In the most advanced models, a rational deposition of alternating cationic and anionic layers—for instance, polyethyleneimine (PEI)–small interfering RNA (siRNA)–PEI–, also permitted developing multilayered nanosystems in which targeting elements could be added in the outermost layer. This design permits placing NAs in middle layers, obtaining additional protection against nucleases. Moreover, this strategy also enables pH-driven cleavage, which permits disassembling the system in endosomal environments, as a consequence of the proton sponge effect associated with polycationic substances [38]. The main drawback of the surface loading strategy is the absence of protection for BMs, which could be deactivated if exposed to blood (opsonization, enzymatic degradation, macrophage-mediated clearance, etc.) [39] or if not properly handled during processing (non-sterile material, accidental contamination, or physicochemical decomposition).

From a protective point of view, the use of pore-expanded MSNs [40] is the most convenient strategy, but only if the cargo is adequately retained until its final destination. To this end, pore sizes should be tuned to allow cargo hosting and, eventually, pore gates may be required [30]. The typical strategies to prepare enlarged-pore MSNs consist of either using large surfactants such as Pluronic or Brij [14] or employing swelling agents able to increase the diameter of the template cetrimonium micelles during the synthesis [41]. Additionally, the use of non-surfactant species, like tannic acid, was also reported as a pore-forming agent [42]. Regarding NAs, it is also important to remark that raw pores of MSNs need to be chemically modified; otherwise, the negative charges of NAs and silanols would undergo a repulsive interaction that would hinder pore loading. This topic was previously visited by us and other groups in previous contributions [20,37]. In the case of proteins and peptides, this effect is not so relevant, as their isoelectric points are always closer to neutrality. In fact, most peptides behave as small molecules and can be loaded satisfactorily in most raw-pore MSNs.

In summary, the loading strategy must be carefully accounted for depending on the carried biomacromolecule. In this way, short peptides can be easily loaded within pores or grafted onto surfaces, while the delivery of bigger and highly charged molecules may suffer from pore rejection if the mesopores are not properly conditioned [43]. Regarding NAs, their outstanding chemical stability permits creating either fancy layered structures or pore-loaded systems [44,45,46]. In addition to typical porous particles, the use of hollow mesoporous silica nanoparticles (HMSNs) also receives interest because of their additional enormous internal space. However, to use them as carriers, their mesopores and surfaces must comply with all requirements outlined for in-pore loading, i.e., sufficient diameter, favorable electrostatic environment, and adequate order to permit effective diffusion processes.

**Figure 2:**
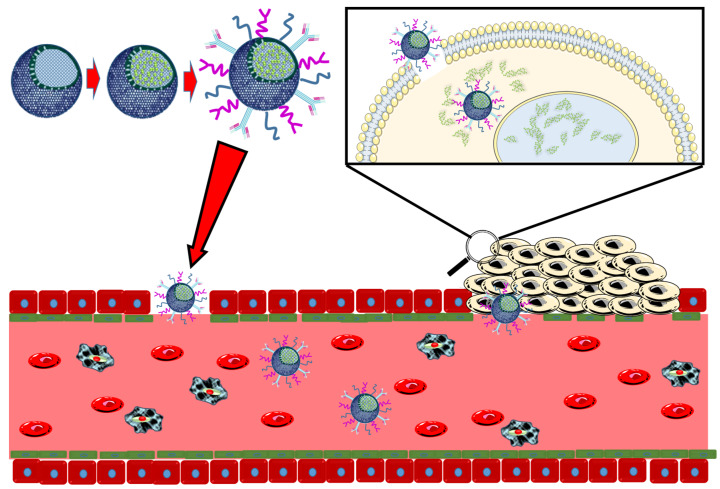
Blood compatibility, colloidal stability, and cell recognition are necessary on therapeutic nanosystems; otherwise, they would not reach their final destination.

In addition to cargo-related modifications, these kinds of nanodevices must also have additional modifications to increase colloidal stability and immune stealth to ensure an adequate trafficking profile (Figure 2). In the case of pore-loaded nanosystems, this can be easily achieved with a common polyethylene glycol (PEG)ylation strategy. On coated nanosystems, it may not be necessary if the particles’ coatings are naturally occurring biomolecules, such as those reviewed herein. In this case, although the integrity of supported molecules is not fully ensured, surface deposition is proven to be a synthetic advantage, as it greatly reduces the number of components and synthetic steps.

## 3. Delivery of Proteins with Therapeutic Effect

**Figure 3:**
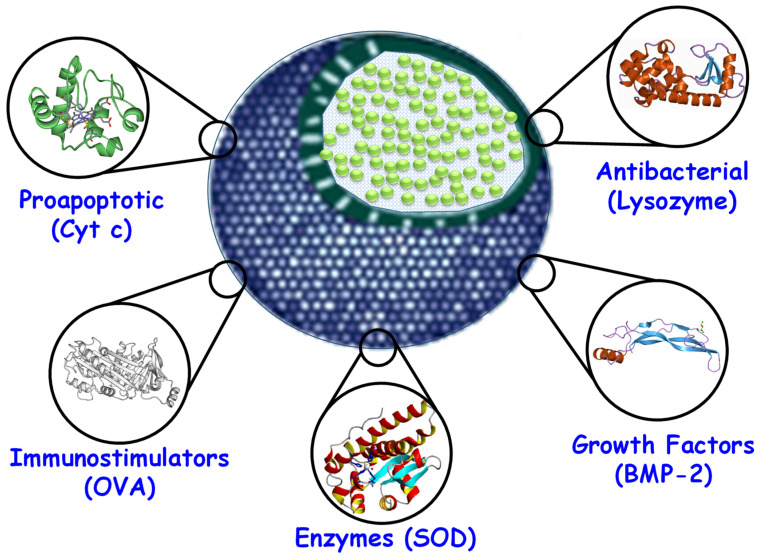
Roles and examples of typical therapeutic proteins delivered with silica-based nanosystems.

As introduced above, the effect and potency of therapeutic proteins rely on their physiological effect and behavior. For example, cytochrome c (Cyt c) triggers a caspase-mediated apoptosis, while immunoglobulins activate the immune system and induce cell destruction. Additionally, certain enzymes and growth factors may be useful to treat certain genetic diseases based on protein malfunction. Therefore, as there is no common therapeutic effect [47], the different approaches are discussed including pro-apoptotic, immunostimulating, enzymes, growth factors, and antibacterial proteins, according to the classification shown in Table 1 and Figure 3. For the interested reader, previously published outstanding revisions dealt with insights into protein loading and delivery with MSNs [48,49,50,51]. 

### 3.1. Anticancer Proteins

Historically, Lin’s group was the first to describe the potential of MSNs to carry and deliver proteins. To achieve this, they focused on Cyt c, a small protein with a pro-apoptotic effect. In their contribution, they pioneered pore-expanded MSNs to allow protein hosting, highlighting the strategy to follow for the intracellular delivery of membrane-impermeable proteins [54]. Herein, they demonstrated that unmodified MSNs with 5.4-nm mesopores were able to host the globular 3.3-nm-width protein to produce effective intracellular delivery, although with no control. To improve this, Griebenow and coworkers evolved a system including a chemical bond able to retain Cyt c inside the mesopores. Their stimulus-responsive system was based on a redox-sensitive disulfide bond that linked Cyt c to thiol-modified mesopores [55], enabling an intracellular, glutathione-mediated release. Shang et al. also employed Cyt c as a model protein for MSN-based delivery, although, in their system, surface adsorption was preferred [56]. In this contribution, the authors did not evaluate the carrier efficiency or the stability of the protein but studied the loading capacity—protein activity—in relation to nanoparticle size. Their results showed that flatter surfaces—larger diameters—permit adsorbing more proteins and, hence, provide higher activities. Another example employing Cyt c was reported by Davis and coworkers, who studied the best-performing linker to connect the protein onto the particles’ surface [57]. In their research, they systematically tested several custom-made linkers against the most critical parameters on optimal delivery for surface-grafted proteins: suitable charging capacity (>40 mV on ξ-potential), cationic character at acidic pH, ability to undergo endosomal escape, and capacity to permanently retain Cyt c immobilized on the surface. They found that MSNs modified with 1 mol.% primary amine were the only material able to satisfactorily accomplish all these tasks, providing a fantastic know-how for subsequent investigations into surface grafting. More recently, Choi et al. explored another possibility to deliver Cyt c, employing eroded MSNs with rougher surfaces and enlarged pores [58]. Herein, these matured MSNs permitted loading the Cyt c instead of obtaining the surface deposition that occurred onto particles bearing conventional (2–3 nm) pores. As a result of the increased in-pore loading, the particles showed an enhanced release compared with both the free Cyt c and the nanosystem with higher surface deposition.

Apart from Cyt c, there were reported numerous anticancer proteins [52,53], albeit few examples in combination with MSNs. A relevant example on the topic was reported with concanavalin A, a lectin with anticancer and antibacterial properties [59,86]. Regarding the anticancer example, the lectin also proved to be an efficient targeting element against human osteosarcoma (HOS) and murine prosteoblast (MC3T3-E1) cells. In our system, MSNs were firstly loaded with the typical chemotherapeutic doxorubicin (DOX) and then coated with a polymeric layer linked to the silica surface through pH-sensitive linkers. To conclude, the concanavalin A (ConA) was grafted onto the surface through amide bonds. The resulting system was able to efficiently deliver the drug intracellularly, but only when the pH dropped enough to cleave the bis-acetal linker. The evaluation of the system demonstrated an enhanced killing effect when both species (ConA and DOX) were co-delivered. This suggested a potent adjuvant effect of the protein, which was able to drop the cell viability from 50% with DOX alone to almost 100%.

### 3.2. Immunostimulating Proteins

Immunostimulation is one of the most popular strategies for improving cancer treatment, as it promises to recruit the patient’s immune system to fight tumors. However, any indiscriminate activation may lead to disastrous results. Therefore, there is interest in developing immunostimulant nanosystems able to induce local immune responses thanks to the enhanced permeability and retention (EPR) effect. MSN-based immunotherapy also has an additional advantage, as these particles were proven to be efficient coadjutants [87,88,89]. Despite these promising features, the delivery of immune-regulating proteins with silica-based carriers is still in its infancy.

Regarding already reported examples, proteins and MSNs can be combined via either surface deposition—grafted or not—or loading into enlarged pores, although those are not the only options. One such exception was the contribution by Lim et al. who delivered immunoglobulin (IgG) to HeLa cells employing a very special silica particle [60]. These were called unconventional perforated HMSNs. Those particles were able to efficiently deliver large membrane-impermeable cargoes, although without any study on immune response. However, their contribution established the basis for local immunostimulation, opening the way to new therapeutic strategies. In more recent examples, several research groups studied immune inductions in mice using nanoparticles. Along this line, Wang, Ito, Tsuji, and coworkers reported a series of articles which firstly studied the immunostimulating behavior of HMSNs [87,88,89] in murine models, and then studied how the surface modification of such HMSNs with the T cell-dependent antigen, chicken ovalbumin (OVA), stimulated the overall response [61]. The authors paid special attention to which markers were upregulated when treating the animals with these devices [62]. They found a four-action pathway: an anticancer effect through the use of HMSNs themselves, an effector memory on the CD4^+^ and CD8^+^ T-cell population, an overexpression on T helper 1 (Th1) and Th2 cytokines, and an enhanced secretion of immunoglobulin antibodies [62]. Employing another silica nanostructure, a multi-shelled dendritic mesoporous organosilica hollow sphere (DMOHS), Yang et al. also obtained similar stimulation patterns when they employed OVA-loaded particles [63]. Herein, a parallel upregulation on CD4^+^, CD8^+^, and Th1 immunoproteins was also reported, characterized by the secretion of interleukin-12 (IL-12), interferon-γ (IFN-γ), and tumor necrosis factor-α (TNF-α). More recently, Cha et al. evolved this system by adding an additional immunostimulating entity. In their model, large-pore MSNs (20–30 nm) were sequentially loaded with OVA plus an additional “danger signal”, an agonist for Toll-like receptor 9 (TLR9) [64]. As a result, an increased immunostimulation was obtained, which again suggests the importance of combined therapies for the development of more effective cancer treatments and vaccines. More recently, these authors implemented their immunostimulant nanosystem by designing a combination of these MSNs embedded in large chemokine-loaded mesoporous nanorods suitable for injection [65]. With this system, the authors overcame the limitations of the intravenous dosage of their previous nanosystem, as, with this approach, they could effectively recruit and mature the dendritic cells in order to achieve a more efficient cancer vaccination. Their final formulation demonstrated a significant tumor progression reduction together with great survival rates.

In addition to the typical strategies to link proteins onto nanoparticles, Niu et al. reported another possible approach for protein delivery. They designed different porous systems with variable cavities by controlling a core–shell assembly between solid silica nanoparticles. In their designs, core particles had a preset size, while encircling particles had smaller but variable diameters. The resulting disposition created different interparticle voids able to host different sized proteins [66]. The resulting nanostructures showed variable roughness (14, 21, and 38 nm, respectively) that permitted satisfactorily loading a wide variety of proteins, such as Cyt c, monoclonal rabbit antibodies (IgG), and even antibody fragments (horseradish peroxidase (HRP)-linked anti-rabbit IgG antibody), preserving in all cases their activities, as demonstrated by surface plasmon resonance. From a therapeutic point of view, the most interesting model was the 38-nm hydrophobically modified rough (non-porous and core-shell) silica nanoparticles (RSNs), which were able to deliver the anti-pAkt antibody into MCF-7 breast cancer cells. In this case, the therapeutic effect occurred because the antibody created a significant reduction of cell proliferation, together with a downregulated expression of the anti-apoptotic B-cell lymphoma 2 (Bcl-2) protein.

In addition to immunogenic proteins, another interesting therapeutic possibility enabled by nanotechnology is non-viral-based vaccination [90,91]. Therefore, this could be achieved if antigens are delivered to immune cells, thereby avoiding a general immune response. One of the first examples of vaccination employing silica nanoparticles was reported by Guo et al., who employed HMSNs to deliver the open reading frame of the porcine circovirus type 2 (ORF2) protein using a murine model [68]. In this case, the nanovaccine was prepared via direct adsorption of the protein onto raw nanoparticles. In mice, the MSN-based delivery of ORF2 induced overexpression of the typical markers: IFN-γ, Th1, CD4, and CD8, suggesting an immune activation. This strategy was also successfully employed to create other specific MSN-based non-viral vaccines for mice: (1) against *Schistosoma mansoni* employing homogenates from the parasite [69]; (2) against porcine enzootic pneumonia by using a recombinant heat-shock protein 70 (HSP70) antigen fragment (HSP70_212–600_) from *Mycoplasma hyopneumoniae* [70]; (3) against enterohaemorrhagic *Escherichia coli* by using a recombinant fragment of filamentous immunogenic protein from enterohaemorrhagic *E. coli* (EspA) protein [71]; (4) against the pathogenic fungus *Paracoccidioides brasiliensis* employing the antigenic protein rPb27 [72]. In light of these results, it is noteworthy to highlight that these nanoparticle-based vaccinations permit immunizing against different types of pathogens, such as parasites, fungi, or bacteria, with a better profile than the free antigen or other known pharmacological formulations in which the delivery toward macrophages is not so efficient.

### 3.3. Enzymes

The deregulation of normal enzymatic levels has a fundamental role in many diseases. For example, toxic or metabolic syndromes are a direct consequence of this protein malfunction. In cancer, the deregulation of homeostasis is also a consequence of an abnormal expression of proteins and enzymes. However, the use of enzymes in therapy clearly goes beyond cancer treatment based on the delivery of cell-damaging proteins. Unfortunately, like all proteins, enzymes usually do not nicely tolerate systemic administration unless properly modified [92]. Such is the case of the p53 anticancer protein capsule designed by Zhao et al. [93] or the collagenase capsule reported by Villegas et al. [94], which performed best only when they had a degradable protective shell. With regard to the enzymes delivered by MSNs, it is important to remark that most examples are located at the surface; otherwise, the diffusion of both substrates and products may be difficult. One of the most studied examples is carbonic anhydrase (CA), which, despite not having a significant therapeutic effect, was widely employed as a model for in-pore loading approaches [73,74]. The advantage of CA as a model is a direct consequence of the facile determination of its remaining activity, as both substrate (CO_2_) and final product (HCO_3_^−^) are small enough to diffuse through the pores with freedom and can be easily determined.

Regarding therapeutic models, the mitigation of toxic syndromes caused by genetic disorders is one of the most promising research fields. One interesting example was reported by Xu et al. [75], who employed MSNs to deliver β-galactosidase (β-Gal) to treat Morquio B syndrome. This disease appears when the enzyme is not able to properly cleave the glycoside bond on oligosaccharides, thus producing the accumulation of substrates. In this case, contrary to other models in which the enzyme was located on the surface, the authors decided to load it into the mesopores to obtain additional protection and long-term stability. In this case, the significant size (119 kDa) of β-Gal made it mandatory to prepare ultra-large-pore MSNs. For this, the authors created core–cone structured MSNs with dahlia-like mesopores that were able to host, preserve, and deliver catalytically active β-Gal to N2a cells. In this case, the large size of mesopores, together with the small sizes of both enzymatic substrates and products, permitted supporting the enzyme within the pores; however, in other cases, in which the substrates have diffusion barriers because of the size or nature, this approach may not be so convenient.

With regard to supported proteins, Mou and coworkers reported the use of two antioxidant enzymes, superoxide dismutase (SOD) and glutathione peroxidase (GPx), to intracellularly deplete reactive oxygen species (ROS). In this case, the different nature of possible substrates demanded an adequate exposure of proteins on the nanosystem; otherwise, they would not be able to prevent oxidative stress. In their first example, the authors developed a mesoporous silica-based multifunctional nanocarrier for SOD [76]. This device employed an Ni^+2^-chelated nitrilotriacetic acid (Ni-NTA)-modified silane, which was able to connect through a histidine moiety to a TAT-containing peptide sequence bound to the enzyme. At this point, the authors denatured the enzyme with urea in order to have only intracellular refolding and to avoid extracellular ROS depletion. The resulting system was able to internalize into HeLa cells and, therein, refold the enzyme into its active form and reduce the oxidative stress induced by paraquat, a well-known superoxide anion generator. More recently, the authors implemented the antioxidant performance of this model by employing two differently loaded nanodevices: one with SOD and the other with GPx [77]. With this combination, the authors found a complementary synergic effect in which the co-delivery of both antioxidant proteins improved the effect of single treatments.

Another interesting example, reported by Han et al., focused on the delivery of human proteasomes to delay tau aggregation associated with Alzheimer’s disease [78]. In this model, the authors employed the same building strategy reported by Mou’s group: Ni-NTA able to bind histidine moieties from active human 26S proteasomes isolated from a HEK293-derived cell line. Again, the system proved to deliver proteasomes without a significant proteotoxic effect associated with its enzymatic activity. At this point, the authors claimed that their system was not intended to permeate the blood-brain barrier, although it could be implemented onto permeable systems, opening the way to novel strategies to prevent and treat Alzheimer’s.

### 3.4. Growth Factors

Growth factors are biomacromolecules responsible for inducing cellular growth, proliferation, and differentiation; they also play important roles in wound healing, bone formation, and vascularization. The most known growth factor is somatotropin, the human growth factor, which is widely employed to promote impaired growth in healthy individuals. The main drawback of this therapy is the need to accomplish a continuous—intramuscular or subcutaneous—dosage in order to achieve a sustained effect. Because of this, it is a treatment with very low adherence and, therefore, it needs improvement. Among the new strategies developed, the most promising are those that permit reaching long-lasting formulations with noninvasive administration routes [95,96]. With regard to the remaining growth factors, mostly cytokines and steroid hormones, it is typical to have compounds with very little aqueous solubility. As a result, they also require form delivery agents to perform their action properly. This need could be a niche for non-biogenic carriers, especially those of high loading capacity such as silica-based platforms.

Regarding the examples available in the literature, ten years ago, Zhang et al. already reported the potential of MSNs as carriers for growth factors [79]. In this contribution, a water-in-oil microemulsion strategy was employed to create ad hoc MSNs for the 18-kDa basic fibroblast growth factor (bFGF) protein. The resulting system was able to release the protein for over 20 days, and it could be satisfactorily taken up by HUVEC cells. Moreover, the cytotoxicity assay showed low toxicity even when administered at high concentrations (50 μg/mL). Despite this promising result, the remaining examples with therapeutic growth factors are limited to two contributions by Gan et al., who employed two complementary approaches for the delivery of the bone morphogenetic protein 2 (BMP-2). In their first contribution, they prepared chitosan-coated MSNs to deposit proteins onto the particles’ surfaces [80], while, in their second work, SBA-15 microparticles with iron oxide nanocaps were employed to load the protein within the 6.2-nm mesopores [81]. In the first example, the authors included dexamethasone, a corticoid, into the mesopores to increment bone growth due to co-delivery of two osteogenic species. Unfortunately, although a significant osteogenesis was achieved, it was completely uncontrolled, and significant ectopic bone formation occurred. To prevent this, the authors changed the model to deliver only the growth factor. To do so, they employed large-pore silica microparticles to carry the BMP-2 and Fe_3_O_4_ nanocaps to prevent a premature release. The resulting pH-driven carrier was able to deliver the protein while exhibiting excellent biocompatibility, but only in vitro, where the huge size of the chosen silica particles did not affect the guest survival.

### 3.5. Antibacterial Proteins

In addition to the previous examples, it is also possible to transport proteins bearing antimicrobial effects. On the delivery of antibacterial proteins with MSNs, most contributions were made with lysozyme, a 14.4-kDa enzyme known to damage bacterial cells by hydrolyzing the major component of Gram-positive bacterial walls, i.e., the β-1,4 bonds between *N*-acetylmuramic acid and *N*-acetyl-d-glucosamine. The first example, reported by Lin et al., employed negatively charged MCM-41 MSNs to bind lysozyme units through electrostatic interactions to provide a stable corona [82]. This model permitted having an antibacterial effect even on Gram-negative bacteria such as *E. coli*. Such an effect was a consequence of the high concentration of lysozyme released in bacterial surroundings. Although forthcoming models improved the antibacterial performance of this device, in this work, the authors set two important advances: the possibility of treating bacterial infections with nanotechnology, and the finding that even Gram-negative bacteria could be highly damaged by these glycoside hydrolase enzymes.

In light of these previous results, Yu’s group attempted to improve the antibacterial capacity by using higher-loading carriers. In one contribution, they differently evaluated dendritic pore MSNs, finding that a bigger pore led to a faster release and, thus, higher antibacterial effects [83]. In the second contribution, they developed silica nanopollens, which are engineered non-porous hollow nanoparticles coated with nanosized silica spikes to provide a rough surface [84]. Similarly, to the former model, they evaluated different roughnesses, finding that a rougher surface led to a better loading and the associated antibacterial effect. A comparison between models seemed to indicate that, in the case of large-pore MSNs, the antibacterial effect of lysozyme was a consequence of a faster release, while, in the case of nanopollens, it was a combination of a more sustained release together with an unknown nanoparticle-induced effect. Unfortunately, these models were only tested on planktonic bacteria. To address a more complex situation in which the bacteria form a biofilm, Ye and coworkers studied hollow MSNs as high-capacity nanocarriers (up to 350 mg per gram in the case of their enlarged pore HMSNs) [85]. In this work, the authors reported a threshold for free lysozyme activity (400 μg/mL), over which there was no therapeutic improvement. However, the use of MSN-based delivery raised that maximal effect, as they showed a more sustained release pattern able to induce a long-lasting effect.

Apart from lysozyme, antibacterial effects with other proteins were also reported such as ConA (in this case, in combination with levofloxacin) [86]. In this example, carboxylate-modified MCM-41 MSNs were functionalized with ConA upon drug loading. Herein, the combined action of both species permitted achieving complete biofilm destruction even at minimal concentrations (10 μg MSNs per mL), improving the precedent models due to combination therapy. In addition to these reported models, there are many more proteins that lead to successful combinations [97]. Such is the case of lactoferrin, known for having antibacterial properties [98], which was extensively employed in the preparation of glioblastoma-targeted nanodevices [99,100].

## 4. Delivery of Peptides with Therapeutic Effect

**Figure 4:**
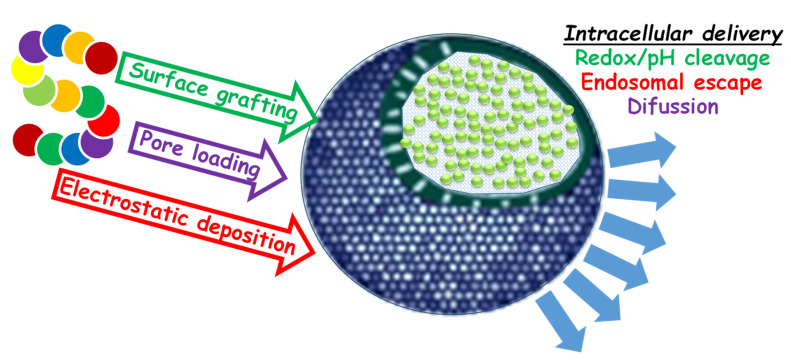
Strategies for delivery of therapeutic peptides employing silica-based carriers.

Many of known bioactive peptides are simplified amino-acid sequences that replicate the active sites of proteins and enzymes. The success of these peptides is a consequence of two complementary aspects: (1) the ability to retain features from their parent proteins, and (2) an outstanding chemical profile that arises from their facile synthesis, significantly low cost, and chemical robustness. Those aspects permitted discovering a large number of peptides of different nature and specificity. For example, regarding targeting, those sequences are selected for their affinity toward eukaryotic [101,102] or prokaryotic [103,104] cell receptors. However, applications of peptides go beyond targeting [105], as they are able to accomplish different tasks in regulating metabolic cycles and signaling. In this section, we focus on the potential of MSN-carried peptides for the development of new therapeutic devices. As shown in Table 2 and Figure 4, their applications range from cancer treatment [106,107,108] to antibacterial effects [107,109], cell regulation processes [110], and immunostimulation.

### 4.1. Anticancer Peptides

Typically, anticancer effects of peptides occur through two possible mechanisms. One of these arises when the peptide is able to disrupt the normal function of the membrane, while the other occurs when it triggers pro-apoptotic pathways. The membrane-disrupting mechanism operates when the peptides are enriched with cationic amino acids: lysine (Lys, K; amine), arginine (Arg, R; guanidine), and histidine (Hys, H; imidazole). Although this mechanism is not fully clear, these peptides demonstrated a certain membrane-lytic effect similar to other cationic species [134]. The first evidence on the topic was observed with TAT-targeted nanosystems [135], as well with other cell-membrane-penetrating peptides [136].

Regarding the use of cationic peptides as therapeutic agents, it is highly desirable to hide their cationic character to prevent premature damages such as hemolysis and embolisms during trafficking. Apart from pore loading, which preserves cargoes from undesired interactions, another interesting possibility arises from the use of additional negative components to balance the resulting surface charge. One elegant strategy, reported by Zhang and coworkers, employed citraconic anhydride to transform an octa-lysine into a negatively charged peptide able to balance the cationic K_8_(RGD)_2_ functionalized MSNs [111]. The resulting outermost layer was able to accomplish three tasks: target integrin receptors through the RDG moiety, show a highly biocompatible neutral charge, and carry a detachable but toxic K_8_ sequence. Unfortunately, the reported studies mainly focused on assessing the effect of carried DOX than on studying the effect of the peptide release, overlooking any potential anticancer effect. In a later contribution by these authors, their system was evolved by including a triphenyl phosphonium group into the KLA peptide sequence (KLAKLAKKLAKLAK) in order to target the mitochondrion. Mitochondria appeared as a key target in cancer treatment because of their roles in regulating cell apoptosis and metabolism [137]. The delivery of antitumoral therapeutic molecules to mitochondria may improve their therapeutic efficacy while avoiding resistance pathways. Furthermore, stimulation of apoptosis mediated by mitochondria could also improve the efficacy of cancer therapy. The resulting organelle-targeted compound was bound to MSNs through disulfide bonds, known to be broken intracellularly by glutathione. Finally, to prevent undesired damages, the authors created a PEGylated anionic polymer shell aimed at increasing biocompatibility and reducing the leakage of topotecan [112]. In this system, the non-loaded model was able to exert a significant pro-apoptotic even at low concentrations, proving the potential of peptides in anticancer therapies; however, as expected, the highest antiproliferative effect occurred when topotecan was loaded within the mesopores.

Encouraged by these results, this group also addressed the synthesis of a multiple pro-apoptotic nanosystem: delivery of DOX, in combination with the delivery of two therapeutic peptides with glutathione-mediated cleavage [113]. In this model, the employed peptides contained different membrane-disrupting sequences, together with two different targeting elements: one of them specific to mitochondrion (C-GKGG-DKLAKLAKKLAKLAK) and the other specific to membranes (C-GRKKRRQRRRPPQ-RGDS). Additionally, to increase the potential cellular damage, the mesopores were loaded with DOX while the peptides were bonded to particles through disulfide bonds, in order to induce oxidative stress upon glutathione depletion. As result, the complete system showed high efficiency against HeLa and COS7 cells, although this effect decreased significantly in the absence of the drug, which highlights the limited effect of membrane-lytic peptides. More recently, this group also employed the glutathione-mediated disulfide cleavage to prepare a drug delivery system for DOX and a membrane-targeted therapeutic peptide rich in tryptophan ((RGDWWW)_2_KC) [114]. In this case, this peptide was designed to have a DNA-intercalant effect due to the high concentration of indoles. This postulate seems to be justified based on the toxicity obtained for such a nanosystem; however, as expected, the best effect was obtained when DOX was co-delivered. More recently, Feng’s group also employed this coating approach to co-deliver DOX and the anticancer peptide KLA. In this model, the final capping was done with a bovine serum albumin (BSA) corona [115], which permitted achieving a double effect: creating a diffusion barrier for both therapeutic agents, and enabling a protease/glutathione-mediated intracellular release. An interesting aspect of the system is that BSA was employed in its wild-type form, which might trigger an additional cellular response when in combination with the remaining multi-apoptotic effects.

Regarding the delivery of peptides that trigger pro-apoptotic routes, the reported examples focused on threading such peptides into the mesopores. For example, Martínez-Máñez’s group reported the use of ε-polylysine as a coating layer to prevent the leakage of the pro-apoptotic C9h (YVETLDDIFEQWAHSEDLK) peptide [116]. In this model, the polylysine coating had multiple roles, as it favored cellular uptake due to its cationic character, while maintaining the C9h peptide within the mesopores until proteases cleaved this protective layer. The in vitro testing of this model proved that the encapsulated peptide showed a better therapeutic profile that its free form. However, this effect reached a maximum plateau over which higher dosages did not augment apoptosis. This points out once again the limited anticancer effect of peptides, which demand additional chemotherapeutic agents to obtain satisfactory results. In another similar example employing pore modifications, Cao et al. reported the delivery of a different proapoptotic peptide. In this model, they employed large-pore MSNs to deliver a bifunctional RGD-containing Hylin a1 peptide (IFGAILPLALGALKNLIK) able to target and kill cancerous cells [117]. To accommodate the peptide, the authors functionalized the internal facets of mesopores to favor threading and enable a pH-dependent release. In vitro studies of this system showed that encapsulation drastically reduced the hemolytic rate shown by the free peptide without affecting the potent cytotoxic effect against HeLa and Hep2 cells in vitro. Published in vivo studies with this system showed a clear tumor growth arrest in murine models, although, unfortunately, complete tumor remission could not be achieved.

As previously outlined, the anticancer effect of peptides and proteins is relatively low; thus, their use is generally limited to therapeutic adjuvants of more active species. However, the overall therapeutic effect may be increased if higher doses can be delivered; in this case, high-loading carriers gain interest. Among silica-based carriers, HMSNs are the most suitable candidates, since they theoretically allow loading in larger quantities than their porous analogues. The diverging aspects of MSNs vs. HMSNs in peptide delivery were studied by Rahmani et al., who focused on pepstatin A, a cathepsin D inhibitor peptide [118]. Surprisingly, the authors found two unexpected behaviors: HMSNs loaded less peptide than typical large-pore MSNs, but their effect was higher. Therein, the authors justified such behavior based on the release patterns observed from HMSNs, which provided a more sustained release (longer therapeutic effect), in comparison to regular pore-expended MSNs, which showed a burst-like release.

Another promising anticancer peptide is NuBCP9 (FSRSLHSLL), which is able to bind the Bcl-2 protein, highly overexpressed in cancer cells, turning a cell protector into an apoptosis inductor [138]. Along this line, Wu et al. reported folate-targeted, large-pore MSNs able to deliver this NuBCP9 peptide into HeLa tumors in zebrafish [119]. The resulting ca. 35-nm-width MSNs with pores in the range of 20 nm showed effective internalization into folate-positive HeLa cells, reaching up to 70% reduction of viability when loaded with the peptide. On the other hand, these nanoparticles showed fantastic biocompatibility, as they permitted obtaining fish survival above 80% in concentrations up to 200 μg/mL. In addition to this targeted example, this peptide was also employed in combination with a typical chemotherapeutic. In this contribution, the authors employed ca. 30-nm MSNs with large pores to load the peptide and an outer coating of a fifth-generation polyamidoamine (PAMAM) dendrimer able to load DOX within the structure [120]. As a result, the nanosystem was able to co-deliver both pro-apoptotic species to several cancer cell lines, achieving almost complete cell destruction in concentrations up to 1 μg/mL. To validate the potential of such a combination, the authors tested the efficiency of their nanosystem against resistant cell lines, obtaining outstanding results except for the case of the DOX-resistant MCF-7 line. Nevertheless, these results must be carefully accounted for, as the outermost PAMAM cationic coating may produce a decrease in overall biocompatibility.

### 4.2. Immunostimulating Peptides

Although most examples of immunostimulation were reported with fully functional proteins, the use of peptides is also possible. Along this line, Xie et al. reported the use of hollow mesoporous silica-based nanocarriers to deliver two melanoma-derived antigen peptides: the hydrophobic H2-K^b^ peptide TRP2_180–188_ (SVYDFFVWL) and the hydrophilic H2-D^b^ peptide HGP100_25–33_ (KVPRNQWL) [121]. In order to achieve the desired double loading, the authors modified their HMSNs with amino groups at the internal space and the mesopores with carboxylates in order to create two different preferential locations for peptides within the HMSNs. Then, to provide adequate retention and colloidal stability, the system was further coated with a lipid bilayer. Furthermore, on this lipid layer, another therapeutic species was included to increase the overall effect: monophosphoryl lipid A (MPLA), a Toll-like receptor 4 (TLR4) agonist. The resulting hollow protocells showed time-dependent uptake by murine bone marrow-derived dendritic cells and, according to data shown, induced cell maturation as seen by the overexpression of CD86, TNF-α, IFN-γ, IL-12, and IL-4 proteins. When the system was tested against melanoma lung metastasis in murine models, the vaccinated animals showed less tumor growth and creation of metastatic lymph nodes, demonstrating that the system was able to induce an effective anticancer response in mice.

### 4.3. Antibacterial Peptides

Although antimicrobial peptides are one of the most promising research lines to fight multi-resistant bacteria, their incorporation into nanocarriers was poorly studied [139]. Among the few systems reported, the contributions made by Braun et al. can be highlighted, who studied the best loading strategy and silica composition for the cationic antibacterial peptide LL37 (LLGDFFRKSKEKIGKEFKRIVQRIKDFLRNLVPRTES). In their first contribution, these authors focused on the membrane interactions between *E. coli* and several silica-based (non-porous, calcined mesoporous, and amino-capped mesoporous) nanoparticles loaded with the LL37 peptide [122]. As expected, the best loading profiles were obtained for the porous calcined—most negatively charged—nanoparticles. In addition, as expected, MSNs provided an adequate protective environment for the peptide and, hence, reduced the associated hemo- and proteolysis. At this point, it is also interesting to remark that these authors also studied how the porous structure affected the delivery, in this case, by comparing regular sized-MSNs with large-pore HMSNs [140]. They found that ca. 2.5-nm-pore MSNs produced a burst release, while the HMSNs showed a more sustained release, in concordance with data obtained by Rahmani et al. [118], suggesting that HMSNs behave better for peptide delivery.

Apart from the activation of immune cells and vaccination, the treatment of infected cells is another big issue that could be solved by applying nanotechnology. One inspiring example was recently reported by Tenland et al., who employed MSNs to deliver an anti-tuberculosis peptide to infected macrophages [123]. In their work, the NZX (GFGCNGPWSEDDIQCHNHCKSIKGYKGGYCARGGFVCKCY) peptide with a proven anti-tuberculosis effect was employed [141]. In this case, the system was assembled by threading the peptide into the mesopores, employing previously optimized nanoparticles [140]. The system demonstrated effective internalization into macrophages and produced peptide release once internalized. As a result, the infecting mycobacterium could the killed without significantly affecting host macrophages; moreover, the MSN-carried peptide showed a longer therapeutic effect than its free form. This effect, seen in other reviewed examples, was justified by the accumulation of nanocarriers within intracellular vacuoles, which created therapeutic reservoirs by maintaining peptide integrity.

Apart from the delivery of antibacterial peptides, combination therapies were also recently reported. Along this line, Zink and coworkers reported the simultaneous delivery of the melittin (MEL, GIGAVLKVLTTGLPALISWIKRKRQQ) peptide and the antibiotic ofloxacin (OFL) with a mesoporous silica-based assembled nanosystem [124]. In this system, the authors employed a very elegant strategy to co-deliver the medium-size peptide together with the small antibiotic. To do so, they prepared large-pore MSNs which were loaded with MEL and capped with a β-cyclodextrin-modified polyethyleneimine and ofloxacin-loaded, adamantane-modified SPION@MSNs capped by cucurbit[6]uril units. The resulting nanoparticles were able to self-assemble and create nanogates able to maintain both drugs within the closed pores. The system was proven to disassemble under magnetothermal induction, thereby releasing both species, which demonstrated efficient destruction of planktonic bacteria and biofilms. Indeed, this system demonstrated its efficiency even when embedded into implants, which were able to prevent bacterial infection in mice.

### 4.4. Growth Factors

As in many other cases, peptides could also have anabolic properties. These features make them interesting candidates for their use as cargoes in new-generation therapeutic devices aimed at tissue growth induction or hormone therapy. The first example in this field, reported by Lin’s group, employed MSNs to intracellularly deliver cyclic adenosine monophosphate (cAMP). To obtain chemical responsiveness, the authors modified the MSNs with a boronic acid –B(OH)_2_ moiety able to coordinate glucuronic acid moieties. The mesopores were then loaded with cAMP and closed through glucuronate-modified insulin nanocaps [125]. The resulting system was sensitive to glucose, as it could be disassembled by chemical displacement. As a result, this system permitted regulating hyperglycemic levels through a dual release mechanism: Insulin to trigger glucose assimilation by liver, and cAMP delivery to induce glycolysis. Unfortunately, this proof of concept was not further evaluated in vivo.

More recently, Sun et al. revisited insulin delivery with MSNs. To do so, they developed a glucose-sensitive nanodevice employing rod-like MSNs and a polymer shell able to change its conformation upon the presence of glucose [126]. In their investigations, the authors optimized the composition of this polymeric shell by using three different monomers: *N*-isopropylaminomethacrylate (NIPAM) to provide the needed morphological shift, 3-acrylamidophenylboronic acid as a glucose-sensitive fragment, and dextran maleate as an optional hydrophilic component. The resulting system could be loaded with insulin at low temperatures (4 °C), in which the polymer adopted an expanded conformation and closed upon heating (37 °C). Both reported compositions showed effective insulin release when glucose was present; however, when pH was below 7, the polymer was not able to shift morphology, avoiding insulin release. Although only in vitro tests were reported, the possibilities of this model are considerable, as the incorporation of dextran may favor colloidal stability and enhance biocompatibility, as suggested by the high cell viability values obtained. Insulin delivery was also investigated by Zakeri-Siavashani et al., who employed SBA-15 mesoporous microparticles [127]; however, therein, the authors profited from the bigger pore size provided by these particles to achieve higher loading and more sustained release. Although their system showed a nice insulin release profile, the typical bigger sizes of SBA-15 particles (generally above 300 nm) limit their application in intravenous formulations, although they may be used to create biocompatible reservoirs.

The facile aggregation of SBA-15 particles caused by their size, which makes their trafficking through alveoli and capillaries extremely difficult, makes these materials ideal candidates for non-systemic therapies, for example, bone replacement and tissue regeneration. In our first contribution to the topic, we found that the osteostatin peptide, derived from parathyroid hormone-related protein (PTHrP_107–111_), could be efficiently delivered using this kind of material [128]. A clear regenerative effect was observed, which permitted recovering bone mass in peri-implant bone regeneration in cavitary defects [129], as well as in osteoporotic subjects [130]. More recently, our investigations focused on the combination of this peptide with osteogenic elements embedded in bioactive glasses [142,143], finding an enhanced bone formation consequence of the exerted combination therapy.

In addition to these examples, other research groups also focused on the combination of osteoinductive peptides with bone-forming agents. Such is the case of the model reported by Mendes et al., which combined an osteogenic growth peptide (OGP, ALKRQGRTLYGFGG) with hydroxyapatite in an SBA-15-based formulation [131]. Therein, the authors found a substantial behavior difference when hydroxyapatite was present, while deeper studies on the possibilities of such a combination were not addressed. Another interesting combination for remineralizing bone was reported by He and coworkers, who employed MCM-41 to prepare a nanosystem able to deliver a BMP-2-derived peptide (KIPKASSVPTELSAISTLYL) together with dexamethasone, a potent synthetic glucocorticoid [132]. Although, in this case, the peptide was not intended to be released, experimental results showed that its presence exerted a clear osteogenic induction. Moreover, the system was able to promote osteogenic differentiation on mesenchymal stem cells in vitro, as indicated by the upregulation of alkaline phosphatase (ALP) and other bone-forming proteins. Additionally, an increased calcium deposition was observed, demonstrating that mesoporous silica nanoparticles are adequate materials for bone engineering.

## 5. Delivery of Nucleic Acids: Gene Modulation and Silencing

Gene modulation and silencing are promising strategies to efficiently treat cancer, as well as cardiovascular or inflammatory-related diseases [20]. NAs are rapidly degraded by endonucleases and barely internalized by cells. Therefore, the biggest challenge today is to develop an effective gene delivery vector to transport and introduce NAs into the gene therapy target cells. These carriers must be easy to manipulate and show non-toxic properties in vitro and in vivo.

There are two main carriers for gene delivery therapy: viral and non-viral vector platforms [144]. For non-pathogenic viruses (retro and adenovirus), the most used platforms are viral vectors, but they present some limitations such as high cost and manipulation, immune response, or inadequate accessibility of genetic cargo internalized by cells [144]. Currently, research groups are focusing their efforts on the improvement of cheaper and easier preparation of non-viral platforms as cation polymers, liposomes, or inorganic porous nanoparticles such as MSNs [145]. In this respect, PEI is a widely used polycation in gene delivery systems due to its amino protonation improving gene delivery [20,144,145]. Liposomes are also commonly applied to enhance the cell internalization of NAs. Both polycations and liposomes present some limitations for therapeutic applications related to their molecular weight or disadvantageous gene escape and physicochemical instability, respectively [20,144,145]. Nevertheless, MSNs showed optimal properties for intracellular delivery of NAs due to their easy manipulation, high loading capacity into the mesopores, surface functionalization, and non-toxicity characteristics [20]. In addition, MSNs can be combined with polycations and other therapeutic drugs for personalized treatment related to cancer and other diseases.

**Figure 5:**
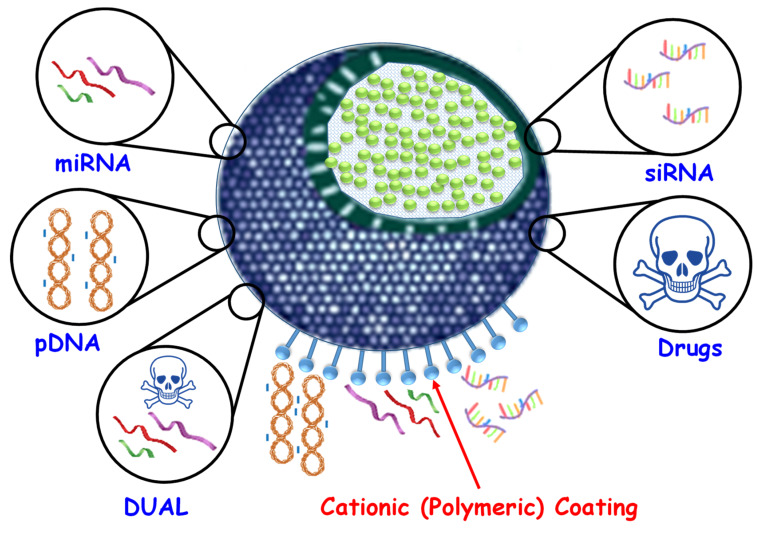
Types of nucleic acids and the possible loading strategies into MSNs for tuning gene expression on cells.

In this regard, plasmid DNAs (pDNAs) are the most widely used gene therapy material to treat different diseases [20,144,145], although there is increasing interest in small interfering RNAs (siRNAs) and microRNAs (miRNAs) for therapeutic application. In this section, we focus on NA-loaded (DNA, siRNA, and miRNA) MSN-based drug delivery systems, as shown in Table 3 and Figure 5.

### 5.1. DNA

As we previously mentioned, MSNs can be functionalized with PEIs for better DNA molecule adsorption and, thus, for a more effective intracellular delivery, protecting NAs from endonuclease degradation due to their positive charge. These positive charges interact with negatively charged cell membranes, inducing higher cell internalization rates and cell death. For example, Zarei et al. [146] developed a functionalized nanosystem based on phosphonate MSNs-PEI loaded with a lysosomotropic factor, chloroquine (CQ), complexed with plasmid DNA. The resulting cell internalization and viability of this pDNA-MSN-PEI was analyzed with the fluorescent protein plasmid (pGFP (green fluorescent protein)), showing a significant increase in transfection of pGFP into the mouse neuroblastoma cell line Neuro-2A. In this respect, Xia et al. [147] proposed the use of a cationic MSN-PEI nanocarrier with a potential therapeutic application. The resulting cationic surface of MSNs facilitated the DNA attachment in order to balance the cationic 10-kDa PEI and, hence, achieve nontoxic effects and higher cellular uptake rates. This nanosystem was loaded with a plasmid DNA (pEGFP) and siRNA construct that was capable of knocking down GFP expression in hepatocellular carcinoma mouse (HEPA-1) cells, with a fluorescent GFP expression of 70% in these cells. In addition, the authors loaded the cationic MSN-PEI nanocarrier with paclitaxel, a hydrophobic anti-tumoral drug used in pancreatic tumor treatment, increasing the cell internalization and delivery of this drug in HEPA-1 cells. On the other hand, Song et al. [29] demonstrated that controlling the nanotopography of MSNs as pDNA vectors had an influence on the transfection efficacy. For example, ambutan-like MSNs-PEI with spiky surfaces showed pDNA-binding capability and a transfection efficacy of 88% in HEK-293T cells, which was a higher rate compared with other MSNs systems. In addition, the authors demonstrated that these types of surface spikes of MSNs induced a continuous open space with two objectives: binding DNA chains through multivalent interactions and protecting the gene cargo covered in the spiky layer against enzymatic degradation, without negative effects on transfection rates. These results indicated that this nanosystem is an interesting approach as a non-viral vector for effective gene delivery.

In a study by Wang et al. [148], an MSN based nanoplatform functionalized with a DNA gate was evaluated in vitro. In this report, the authors developed a fluorometric method for adenosine triphosphate (ATP) recognition using rolling circle amplification (RCA) consisting of proximity ligation-mediated amplification. In addition, the nanosystem was functionalized with graphene oxide modified with folic acid (FA) as a DNA vehicle and loaded with DOX, to evaluate the control release efficacy in HeLa tumoral cells. Following the RCA process, long DNA chains that contained a complementary strand to the DNA at the gate permitted dehybridizing such a nanogate and producing DOX release into tumoral cells. This nanoplatform was effectively internalized by the FA receptor, upregulated in those tumoral cells, while the DOX release induced increased cell toxicity compared with control systems, improving the targeted cancer therapeutics on these HeLa cells. In another study by Wang et al. [149], a DNA-capped MSN nanocarrier loaded with DOX for tumor marker-triggered on-demand drug release was developed and evaluated in vitro. As a first step, a DNA biomolecular gate adsorbed on the MSN–NH_2_ was developed at neutral pH via electrostatic adsorption. In the absence of a stimulus, the pores were locked, and it was not possible to release the drug. However, when the nanosystem recognized the internal stimulus (survivin messenger RNA (mRNA) or miR-21, overexpressed biomarkers in cancer), the DNA caps were removed from the MSNs, allowing DOX release. This stimulus-responsive behavior was confirmed by different microscopy techniques in cell-acute myeloblastic leukemia (HL-60) cells. This nanocarrier was effectively internalized by these cells, releasing DOX cargo into the cytoplasm and inducing significant cell death. Therefore, this nanoplatform is a very useful novel system for both imaging diagnosis and therapeutic controlled drug delivery applications. In this regard, Li et al. [150] designed and evaluated another anti-tumoral and gene drug co-delivery nanosystem with on-demand release properties in vitro and in vivo. For this purpose, the authors developed dendritic MSNs modified with imidazole groups employing a Schiff-base imine linkage, which permitted loading DOX within the pores and electrostatically depositing the survivin short hairpin RNA (shRNA)-expressing plasmid (iSur-pDNA). The imidazole functionalization triggered efficient endosomal escape, improving the accumulation of iSur-pDNA and gene knockdown efficacy. Such nanosystems were successfully internalized by QGY-7703 hepatoma cells and decreased tumoral cell viability in vitro, due to the pH-sensitive co-delivery of DOX and iSur-pDNA. In addition, this nanoplatform induced tumor growth reduction in H-22 tumor-bearing mice, indicating that it could be a promising nanocarrier for co-delivery cancer treatment.

On the other hand, aptamers are short single-stranded oligonucleotides with high affinity and specificity to several molecule targets, and they exhibit potential properties as therapeutic and diagnostic factors in different diseases [176]. In this regard, Sun et al. [151] developed an MSN-based nanocarrier with different DNA molecules on the surface for self-assembly. For the controlled delivery of anti-tumoral drug cargo (DOX), the authors used an aptamer oligonucleotide as a gatekeeper and other oligonucleotides on the MSN surface to allow DNA-guided immobilization bearing single-stranded capture oligonucleotides. This nanovalve induced an increase in cell adhesion rates in MCF-7 adenocarcinoma cells, with an efficient triggered release of DOX drug in these cells. The DNA-directed self-assembly aptamer-based nanosystem is efficient in surface-bound monolayers and can be used as a delivery system for different applications and treatments, allowing site-specific delivery of anti-tumoral drugs. In the same way, Li et al. [152] developed a dual multi-locked DNA valve HMSN-based nanosystem to intracellular cancer-related mRNAs for controlled DOX drug release. The upregulated endogenous targeted mRNAs (K1 and GalNAc-T mRNAs) were able to dehybridize the multi-locked DNA valves and open the pores to release DOX. As a result, a synergic effect was obtained, caused by the intratumoral delivery of the drug together with the depletion of TK1 mRNA, implicated in cell division as a tumor growth factor, and GalNAc-T, upregulated in several tumoral cells. The nanocarrier was evaluated in vitro in different cancer cells, inducing higher rates of cytotoxicity compared to non-tumoral cells. In an in vivo study, Pascual et al. [153] designed another novel non-viral drug delivery and diagnostic nanocarrier based on a capped MSN-NH_2_ hybrid platform, which was loaded with DOX and gated with Mucin 1 (MUC1) aptamer (S-apMUC1). MUC1 is a protein present in the cell surface and upregulated in breast cancer cells. The S-apMUC1-MSNs nanosystem was efficiently and specifically internalized only by tumoral MDA-MB-231 cells related to MUC1 receptor overexpression of these cells. After S-apMUC1-MSNs internalization by tumoral cells, the DOX cargo was released into the cytoplasm of tumoral cells, inducing a decrease in cell viability. This nanoplatform exhibited reduced cargo release when DNAse I was not present. In vivo, S-apMUC1-MSNs were radionuclide-labeled with ^99^Tc for a radio-imaging study in MDA-MB-231 tumor-bearing Balb/c mice. S1-apMUC1-Tc displayed significant tumor signaling and accumulation in these mice. These results suggest that MSN nanosystems capped with aptamers showing radiopharmaceutical properties are promising hybrid nanomaterials in the clinical context.

Of particular interest is a study by Srivastava et al. [154], where the authors proposed a novel telomerase-responsive delivery of DOX loaded in an MSN nanosystem against telomerase-positive tumoral cells. DOX loading was performed using a telomeric repeat complementary sequence and a telomerase substrate primer sequence, allowing controlled release in MCF-7 cancer cells. This functionalized nanocarrier was efficiently internalized, and it significantly reduced the tumoral cell viability, with an inhibition of function when a specific telomerase inhibitor was present. This oligo-wrapped nanoprobe specific for telomerase was used in a telomerase-positive Dalton’s lymphoma mice model. The nanosystem induced a significant inhibition of tumors, enhanced survival, and re-established histopathological parameters, including neo-angiogenesis.

Furthermore, DNA vaccination was proven to provide an immune response to viruses or infectious diseases [177]. In this regard, Wang et al. [155] developed a layered double hydroxide (LDH) MSN nanocarrier as a vaccine delivery platform and immune stimulant, using a GFP expression plasmid as model DNA. The pDNA-MSN-LDH nanosystem showed high internalization rates in monocyte-derived dendritic cells (MDDCs) and stimulated macrophage activation via nuclear factor kappa B (NF-κB) signaling pathway, increasing IFN-γ, IL-6, CD86, and major histocompatibility complex class I (MHC-I). In vivo, the nanocarrier immunized BALB/c mice, indicating that the DNA vaccine-MSN-LDH system increased the serum antibody response and promoted T-cell proliferation, skewing T helper to Th1 polarization. Taken together, these results indicated that MSN-LDH nanosystems could operate as a potential non-viral gene delivery platform.

### 5.2. siRNAs

Small interfering RNA (siRNA) are highly potent, short, double-stranded RNA molecules (20–25 bp) used in gene-based therapy of different diseases that can be delivered in a sustained manner [178]. Moreover, siRNAs are anti-cancer targets because they can modify the expression of pro-apoptotic oncogenes and cell-cycle key regulators. However, siRNAs have a very short half-life and poor cell internalization capability [178]. In this regard, MSNs attracted pronounced consideration for the intracellular delivery of NAs due to their high loading capacity into the mesopores and surfaces. Polycations such as PEI combined onto the surface of MSNs permit a more stable encapsulation, loading, controlled release, and intracellular internalization of potential siRNA cargoes [178]. In addition, positively charged amine-rich nanosystems are required for a better result in siRNA/MSN cellular uptake rates [178]. Future in vivo efforts are necessary to validate the tissue-specific siRNA therapeutic efficacy using imaging techniques to analyze MSN biodistribution at the whole-body level. On the other hand, it is mandatory to find nanosystems able to efficiently deliver those therapeutic siRNAs in vivo with enough efficiency, as well as with protective features against nucleases, able to enhance their gene-silencing capabilities at the target tissues.

In this respect, Shen et al. [156] developed a universal siRNA multi-component system carrier based on MSNs functionalized with cyclodextrin-grafted polyethyleneimine (CP). CP offers a positive charge to improve the siRNA loading on MSNs through electrostatic interaction, allowing endosomal escape. To analyze the effectiveness of the proposed delivery system, an siRNA of the M2 isoform of the glycolytic enzyme pyruvate kinase (PKM2) was used, due to it being overexpressed in several cancer types. Firstly, siRNA-loaded CP-MSNs were positively evaluated in vitro in MDA-MB-231 human breast cancer cells with higher rates of cellular internalization and gene silencing capability. The nanosystem showed specific tumor accumulation and gene silencing in an orthotopic mouse model of MDA-MB-231 cells, inhibiting tumor cell growth, invasion, and migration. Prabhakar et al. [157] designed a new nanosystem based on MSNs with expanded mesopores and pore–surface-hyperbranched PEI tethered with redox-cleavable organic linkers that could carry high concentrations of siRNAs. This stimulus-responsive nanosystem was highly internalized by MDA-MB-231 cells, releasing the cell-killing siRNA as cargo into the cytoplasm, followed by endosomal escape triggered by the intracellular redox situation, with high efficiency in long-term gene knockdown for several days. In addition, long-term stability of therapeutic nanosystems is mandatory to provide personalized medicine. In this respect, lyophilization or freeze-drying are useful and common methods to ensure this aim. Ngamcherdtrakul et al. [158] successfully reported a lyophilized nanosystem consisting of MSNs modified with cationic polymers (PEI), PEG, and antibody (trastuzumab), which was loaded with a human epidermal growth factor receptor 2 (HER2) siRNA upon reconstitution. This nanosystem was successfully internalized by HER2^+^ human breast cancer cells, BT474, decreasing their viability; it maintained a cake-like structure and retained hydrodynamic size, charge, siRNA loading ability, and silencing efficacy. With the tested method, the lyophilized nanocarrier can be stored stably for eight weeks at 4 °C and at least six months at −20 °C.

Oral cancer is the seventh most frequent cancer and the ninth most frequent cause of death in the world [179]. About 90% of oral cancer is of squamous cell carcinoma type (SCC) [179]. Currently, surgery and radiation, combined or not with chemotherapy, are the main treatments, but this approach presents several limitations due to multidrug resistance; thus, new therapeutic drugs must be evaluated. Lio et al. [159] developed a topical formulation for the transdermal delivery of siRNA built on MSNs for the treatment of skin disorders. In this regard, transforming growth factor beta receptor I (TGFβR-1) is overexpressed in patients with skin SCC, and the anti-tumor effects of TGFβR-1 inhibitors were proven in several types of cancer. The nanosystem was built by loading TGFβR-1 siRNA into the pores of MSNs and further coating the resulting particle with a layer of poly-l-lysine (PLL) to improve transdermal delivery. The tested platform demonstrated positive cellular uptake rates and detection of specific gene biomarkers in human SCC cells in vitro. This system induced a topical delivery of TGFβR-1 siRNA targeting the SCC in a mouse xenograft model, decreasing the TGFβR-1 siRNA levels and reducing tumor volume. These results indicated that this platform is a promising non-invasive transdermal drug delivery system. Moreover, multidrug resistance plays an important role in the failure of oral cancer chemotherapy. In this sense, in a study by Wang et al. [160], a novel MSN loaded with both multidrug resistance protein 1 (MDR1) siRNA and DOX was evaluated in vitro and in vivo to directly kill tumor cells without the effect of multidrug resistance. MSNP-PEI@siRNA loaded with DOX was efficiently transfected into human oral squamous carcinoma DOX-resistant (KBV) cells, decreasing the gene expression of MDR1 and increasing the apoptosis of these cells. This nanocarrier significantly reduced the tumor size and decelerated tumor growth rate in vivo, showing a huge potential therapeutic application for multidrug-resistant cancer. On the other hand, Shi et al. [161] developed hyaluronic acid (HA)-assembled MSNs nanosystems loaded with MDR1 siRNA and protein MutT homolog 1 (MTH1) inhibitor TH287. Downregulation of MTH1 protein expression increased oxidative stress levels and significantly decreased tumor survival and proliferation. TH287 is a potent small molecule that inhibits the MTH1 protein, and it could act as a potential chemotherapeutic drug. The HA-MSNs nanosystem was successfully internalized by CAL27 cancer cells, inducing a high rate of cell apoptosis when TH287 and MDR1 siRNA were present. In vivo, this nanocarrier reduced the tumor growth compared with untreated control and free TH287 in male Balb/c mice. These results suggest that HA-MSN-based nanosystems are a promising vehicle to control the delivery of MDR1 siRNA and TH287, with a possible combination with other therapeutic agents for oral cancer treatment.

Regarding multidrug resistance as a persistent limitation for cancer treatment, Pan et al. [162] proposed a novel pH-responsive delivery nanosystem based on carboxylated MSNs (MSN-COOH) with a zeolitic imidazole framework-8 (ZIF-8) film synthesized for pore blocking and efficient loading of anti-apoptotic B-cell lymphoma 2 (Bcl-2) siRNA. The nanosystem developed was efficiently internalized by MCF-7/ADR and SKOV-3/ADR tumoral cells. The ZIF-8 film can be degraded in the acidic endo-lysosome and trigger the intracellular release of both siRNAs and potential antitumor drugs, decreasing cancer cell proliferation. In this regard, Choi et al. [163] developed a new biodegradable and biocompatible amine-free, neutral MSN-based nanocarrier for siRNA delivery. In this study, Bcl-2 siRNA was loaded via a calcium ion (Ca^2+^)-mediated interconnection between phosphates of siRNA and surface silicates of MSNs, reducing the risk of amine-related cytotoxicity and immunogenicity. This nanosystem showed positive results in internalization rates in tumoral SKOV-3 cells, inducing a significant decrease in cell viability when the siRNA was released, compared with MSNs or siRNA alone. This reduction in tumoral cell proliferation was dramatically enhanced when the nanocarrier was loaded with DOX. The therapeutic potential of the co-delivery system was also analyzed in SKOV3 xenografts in nude mice. The authors found that co-delivery of Bcl-2-siRNA and DOX MSNs-Ca^2+^ reduced tumor volume with an inhibition rate of 72%, higher than MSNs-Ca^2+^ loaded with DOX and MSNs-Ca^2+^ with siRNA. The results indicated that Ca^2+^-bound MSNs can act as an siRNA nanocarrier with a less toxic, more effective, and safer siRNA potential clinical application. On the other hand, patients with epithelial ovarian cancer (EOC) metastases relapse after initial effective chemotherapy treatment due to multiple drug resistance. In order to resolve this problem, Shahin et al. [164] proposed a novel nanoparticle delivery platform to reverse chemoresistance based on HA-MSNs loaded with TWIST siRNA. Hyaluronic acid (HA) is a ligand for CD44, which is overexpressed and correlates with worse prognosis in EOC. TWIST is a key protein implicated in epithelial–mesenchymal transition, cancer metastasis, angiogenesis, and drug resistance. The developed nanocarrier was successfully internalized in vitro by F2 and Ovcar8 cell lines, inducing a sustained TWIST gene knockdown. MSN-HA@siRNA showed safety and specific tumor targeting in an EOC mouse model. Mice treated with the complete nanosystem induced significant tumor reduction, and, when the authors combined this nanocarrier with cisplatin, the reduction of tumor growth was greater compared with the corresponding control groups.

For the metastatic spread of cancer, growth of the vascular network is a critical event [29]. Vascular endothelial growth factor (VEGF) is a glycoprotein and key mediator of angiogenesis in tumoral cells [180]. Keeping this in mind, Zheng et al. [165] proposed a new nanocarrier based on MSNs functionalized with FA-targeted tumoral cells and co-loading of VEGF siRNA and ursolic acid (UA), a pentacyclic triterpenoid with anticancer efficacy. HeLa (FA receptor-overexpressed) and HepG2 tumoral cell lines were used to analyze the MSN-FA-UA@siRNA nanosystems in vitro. The MSN-FA nanocarrier enhanced transfection efficiency and siVEGF stability, improving the targeted antitumoral efficacy of siVEGF and UA, with a significant decrease in cancer-related VEGF protein levels in HeLa cells. In this sense, the same authors [166] developed an alternative MSN nanosystem consisting of an asialoglycoprotein receptor (ASGPR)-targeting drug delivery system for co-delivery of siVEGF and sorafenib (SO), a multi-kinase inhibitor that can target VEGF for the inhibition of tumor growth and neo-angiogenesis in hepatocellular carcinoma (HCC). Lactobionic acid (LA) is derived from lactose oxidation, and it was able to effectively target human HCC. In addition, ASGPR is overexpressed in several types of cancer cells, facilitating hepatic infection, and it is specifically recognized by glycoproteins which bind with it. The MSN-LA-SO@siVEGF nanocarrier induced synthesis (S) phase cell-cycle arrest, increasing the anti-cancer efficacy (cell apoptosis) of SO and siVEGF through the active targeting of LA in ASGPR-overexpressing Huh7 tumoral cells. Furthermore, this nanosystem enhanced the siVEGF transfection efficiency and inhibited the expression of angiogenesis-related VEGF proteins in these tumoral cells. Taking both studies together, these types of functionalized MSN-based nanosystems could be potential nanocarriers for targeted delivery of anti-tumoral drugs and VEGF siRNAs to improve anti-tumor capability.

On the other hand, fibrotic diseases are related to augmented oxidative stress and upregulation of pro-fibrotic genes [181]. Oxygen species (ROS), nicotinamide adenine dinucleotide phosphate (NADPH) oxidase 4 (NOX4) and heat-shock protein 47 (HSP47) are implicated in this disease [181], producing disproportionate collagen synthesis. Morry et al. [167] proposed an MSN platform with PEI and PEG coating that effectively delivered HSP47 siRNA in vitro and in vivo. This nanocarrier induced a knockdown of the HSP47 gene and a decrease in ROS and NOX4 levels in an in vitro model of fibrosis based on TGF-β-stimulated fibroblasts. Intradermal administration of MSNs@siHSP47 successfully decreased HSP47 protein levels in a bleomycin-induced scleroderma mouse model in vivo, reducing ROS production and different pro-fibrotic markers. These nanocarriers could be a very interesting platform to treat both fibrotic and inflammation diseases. On the other hand, it is necessary to develop anti-fibrotic drugs for chronic liver fibrotic diseases. Hepatic stellate cell (HSC) activation is present in the progression of liver fibrosis, secreting extracellular matrix proteins as tenascin-C (TnC). Vivero-Escoto et al. [168] proposed an MSN@siTnC based nanosystem as a favorable alternative to evaluate the siRNA therapy of chronic liver disease in preclinical trials. This novel nanocarrier was successfully internalized by human stem cells in vitro and produced a downregulation in TnC mRNA and protein levels, inducing a decrease in inflammatory cytokine expression by macrophages and hepatoma migration. These promising results must be tested in a liver fibrosis in vivo model.

As demonstrated in this section, siRNA-MSN based nanosystems were widely studied and showed a high efficiency in silencing genes and reducing tumoral growth related to cancer processes in vivo. Nevertheless, in tissue engineering applications, scaffold-mediated delivery is desired to achieve local and sustained release of drugs. In this respect, Pinese et al. [169] developed an MSN-PEI@siRNA nanocarrier delivered from electrospun scaffolds via surface adsorption and nanofiber encapsulation. These nanosystems were either combined with the surfaces of nanofiber substrates or directly encapsulated. MSN-PEI@siRNA coated scaffolds offered sustained availability of siRNA for at least 30 days and five months in the case of MSN-PEI@siRNA-encapsulated scaffolds within the electrospun fibers in human dermal fibroblasts cells in vitro. The scaffolds showed excellent bifunctionality in vitro and in vivo by targeting collagen type I (COLL1A1) as a target gene to reduce fibrous capsule formation. These platforms were subcutaneously implanted in a rat model in vivo and COLL1A1 siRNA/MSN-PEI induced a reduction of 45.8% in fibrous capsule formation after four weeks compared to negative siRNA treatment. This method improved siRNA internalization rates and sustained targeted protein silencing in vitro and in vivo. The nanoplatform proposed by these authors can be used to release and internalize siRNA/miRNA combined with other specific low-molecular-weight drugs in long-term tissue engineering applications and cancer. In addition, the current pharmacological therapy of bone diseases such as osteoporosis presents several limitations related to bioavailability and toxicity. In this sense, gene knockdown through siRNA delivery received great attention as a promising treatment in osteoporosis [182]. In a recent study by Mora-Raimundo et al. [170], a nanosystem based on MSN-PEI, which can effectively deliver SOST siRNA and osteostatin inside cells, was evaluated in vitro and in vivo. The SOST gene inhibits the Wnt signaling pathway, decreasing osteoblast differentiation, whereas osteostatin is a pentapeptide with osteogenic and anti-osteoclastic properties [183]. SOST siRNA/MSN-PEI loaded with osteostatin induced a dramatic suppression of SOST gene expression in mouse embryonic fibroblastic cells with the consequent upregulation of several osteogenic markers with a synergistic effect. In vivo, the complete nanosystem was injected in the femoral bone marrow of ovariectomized mice, and the results were in concordance with in vitro studies, knocking down SOST and upregulating the osteogenic marker levels in a synergistic manner. The nanoplatform proposed by these authors was able to transport, co-deliver, and internalize SOST siRNA and osteostatin in cells, preserving its activity and achieving an effective silencing effect.

### 5.3. miRNAs

MiRNAs are small endogenous noncoding RNA molecules (17–25 nucleotides) that act as regulators of gene expression and downregulate target proteins via miRNA degradation or translational inhibition [184]. The aberrant expression of miRNAs in several cancers is associated with several mechanisms such as cellular differentiation, proliferation, survival, and apoptosis [184,185]. MiRNAs can accomplish a gene knockdown effect by regulating multiple miRNAs compared to siRNA, which is very useful when treating complex multifactorial diseases such as cancer [184,185]. Several drugs based on miRNAs were used in clinical trials in cancer [186]; however, the effective and safe delivery of anti-miRNAs or miRNA mimics is a key challenge for clinical applications.

Regarding these objectives, Li et al. [171] proposed a novel nanosystem based on MSN functionalized with polymerized dopamine (PDA) and AS1411 aptamer loaded with microRNA-155 (anti-miRNA-loaded MSN@PDA-Apt) for the specific treatment of colorectal cancer (CRC). Upregulation of miR-155 (oncogenic microRNA) was found in several cancer-related pathways, including in CRC. Firstly, the authors demonstrated that nuclear factor kappa B (NF-κB), an important transcription factor with a crucial role in the process of tumor growth, has a positive feedback loop with miR-155 in vitro and in CRC tissues. MSNs-anti-miR-155@PDA-Apt decreased miR-155 expression in SW480 cells and successfully targeted the CRC tumor, leading to gene knockdown and tumor growth reduction, thanks to both active targeting of AS1411 aptamer and passive targeting of the EPR effect. In addition, the same authors [172] explored the miR-328 pathway and developed a new pH-responsive nanoplatform consisting of MSNs-miRNA-328 decorated with PDA, a cell adhesion molecule aptamer, and bevacizumab (MSNs-miR-328@PDA-PEG-Apt-Bev) for the dual-targeting treatment of CRC. MiR-328 is a tumor suppressor downregulated in several human cancers, including CRC, and it is correlated with drug resistance. The authors demonstrated for the first time that CPTP, a ubiquitously expressed lipid transfer protein associated with inflammation and CRC, is a direct target of miR-328. MSNs-miR-328@PDA-PEG-Apt-Bev increased miR-328 levels and inhibited CPTP expression in SW480 cells, increasing binding ability and showing much higher cytotoxicity in vitro and in vivo. In addition, this nanoplatform showed efficient gene silencing and tumor growth inhibition of the target tumor in a SW480 xenograft mouse tumor model in vivo. Taken together, these results indicate that the MSN-based functionalized nanoplatforms reported herein show promise for miRNA therapy in cancer, specifically CRC. In a recent study by Ahir et al. [173], a nanosystem based on MSN-HA@miRNA with an appended PEG–poly(lactic-*co*-glycolic acid) (PLGA) polymer to target triple-negative breast cancer (TNBC) was evaluated in vitro and in vivo. In this context, miR-34a is downregulated and miR-10b is upregulated in TNBC disease, inducing tumorigenesis and metastatic dissemination. These authors proposed the MSN nanocarrier for co-delivery of miR-34a-mimic and antisense-miR-10b, targeting the CD44 receptor. In vitro, this nanosystem showed positive results in terms of cellular internalization rates, release profile, and a subsequent pro-apoptosis effect in human mammary carcinoma cell lines (MDAMB-231 and MDAMB-468). In vivo studies exhibited a high specificity in TNBC tumor targeting, leading to effective tumor growth reduction and metastasis delay in mice.

As previously mentioned, MSN nanosystems emerged as a promising bone regeneration methodology in bone tissue engineering. In this regard, Yan et al. [174] developed a novel miR-26a delivery nanosystem based on MSNs. MiR-26a was confirmed to regulate several pathways of osteogenic differentiation and promote bone regeneration. The authors demonstrated the protection effectiveness of the vectors to the miRNA and the positive internalization rates in rat bone marrow stromal cells (BMSCs) in vitro, releasing miR-26a into the cytoplasm without cytotoxicity effects. This nanosystem promoted stem-cell osteogenic differentiation, inducing an increase in alkaline phosphatase activity and mineralization, as well as in several genes and proteins implicated in osteogenesis in BMSCs. The nanocarrier proposed by these authors provides new methods and strategies for the delivery of mRNAs in bone tissue engineering, but it is necessary to further validate them in vivo.

On the other hand, a combined siRNA/miRNA therapy would be a very interesting approach, especially for cancer treatment, targeting multiple disease-related pathways and silencing specific genes. Currently, the use of this combination in clinical studies shows several limitations such as the availability of safe and effective systemic delivery nanocarriers with efficient tumor penetration. In this respect, Wang et al. [175] developed multifunctional tumor-penetrating MSNs for co-delivery of siRNA (siPlk1) and miRNA (miR-200c), upregulated in several types of tumor and implicated in cancer development and progression. In addition, the authors functionalized the nanosystem to facilitate endosomal scape using a photosensitizer indocyanine green (ICG) and surface conjugation of the iRGD peptide to allow deep tumor penetration. The complete nanosystem induced an increase in internalization rates in MDA-MB-231 cells and three-dimensional (3D) tumor spheroids in vitro, whereas ROS produced by ICG upon light irradiation induced the endosomal scape of the siRNA/miRNA into the cytoplasm with a deleterious effect on cell viability. In vivo, a significant reduction in tumor growth and metastasis upon short-light irradiation was found when the nanosystem was intravenously administrated in mice with metastatic breast cancer.

## 6. Delivery of Glycan-Based Biomolecules

Beyond the use of proteins, peptides, and nucleotides, glycan-based structures were also successfully employed in MSN-based delivery. Among them, the most recurrent examples are reported with heparin, chondroitin sulfate (CS), chitosan, and HA. Those compounds, apart from being important components of structural matrices, are known to affect many biochemical processes. For instance, HA is a valuable targeting element for the development of new therapeutic tools, as it is able to interact with CD44 and CD168, which are upregulated in many cancerous cells and are closely related to cell adhesion and migration in metastases [187,188].

Similarly, chitosan [188,189,190] and chondroitin sulfate [191,192,193] were employed for the development of high colloidally stable and biocompatible nanosystems with certain targeting capabilities. Moreover, as both substances are important structural components of extracellular matrices on vertebrates and crustaceans, they can act as bioactive components for tissue regeneration. Such was the case in the contributions by Kavya et al. [194] and Porgham Daryasari et al. [195], who employed those elements to induce osteogenesis through two different approaches. In the first approach, Kavya et al. designed a crosslinked chitosan/CS scaffold reinforced with nanometric SiO_2_. In this system, the presence of silica permitted improving the mechanical properties and obtaining slower degradation, while the glycans permitted obtaining an adequate moisture swelling for creating a favorable environment for cell proliferation [194]. In the article by Porgham Daryasari et al., the authors employed chitosan-coated, dexamethasone-loaded MSNs as a bioactive component in a poly-l-lactic acid scaffold. The system was proven to induce osteogenic differentiation of human adipose stem cells according to the ALP activity [195]. Although both models offered promising results, the absence of osteoregeneration experiments in vivo makes these materials a mere design exercise, in which the presence of glycans is not completely justified. Apart from its use as a stabilizer, CS was also successfully employed as a functional coating able to interact with blood lipoproteins through a double effect: electrostatic interaction plus saccharide recognition [196]. Although these CS-modified dendritic MSNs permitted selectively binding and isolating low-density lipoproteins (LDL) for analytical purposes, the potential of such a nanosystem as a therapeutic agent is of enormous interest, as it may enable an interesting therapeutic effect against hypercholesterolemia and blood vessel atheromatosis.

Heparin is a highly sulfated, negatively charged glycosaminoglycan with outstanding properties as an anticoagulant. Beyond injectable applications, heparin is employed as an anticoagulant surface in medical devices like test tubes and dialysis machines. Regarding additional uses in combination with mesoporous silica, its capacity as a targeting moiety was also described against human hepatocyte carcinoma HepG2 [197,198] and HUVEC cell lines [198].

Additionally, as a consequence of its anticoagulant effect, some investigations focused on improving its release profile from silica-based carriers as nanodepots. Along this line, the research articles authored by Zhu and coworkers performed two systematic studies on how different types of mesoporous silica, with varying pore sizes and functionalization, affected the loading-release process [199,200]. Based on their results, the authors established that a slight pore enlargement of MCM-41 yielded better adsorption and release. Indeed, they also demonstrated that the presence of amino groups in the mesopores increased the amount of heparin adsorbed, albeit at the expense of slowing down the release [199]. With regard to SBA-15, they found that, apart from pore size and charge, the presence of rough pore surfaces caused by carbonaceous deposits upon calcination boosted both the retention and the release of heparin, improving the performance of such a material and enabling access to better-performing nanodepots.

Apart from its use as a drug delivery agent, there are examples of heparin-loaded silica-based materials used as anticoagulant coatings for medical devices. For example, Wei et al. reported the use of SBA-15 to prepare a heparin-releasing anticoagulant coating [201]. Their system employed amino-modified mesopores to favor heparin loading and delay its release. To incorporate these particles into chosen substrates (silicon wafer, glass, or polyvinylchloride), the authors coated the particles with polydopamine and the substrate with a catechol-modified chitosan polymer. This configuration permitted depositing the negatively charged, heparin loaded MSNs onto chitosan, which was further bound to the catechol–dopamine coating of substrates. Finally, to prevent undesired detachment, this dopamine–catechol bond was oxidized with NaIO_4_ in order to create a non-degradable polymeric layer. When the authors tested this coating against blood, they found a very low fibrinogen adsorption, as well as platelet adhesion and hemolysis, which is in concordance with a sustained anticoagulant release and good biocompatibility. More recently, Wu et al. reported a similar approach but with an added feature: antibacterial adhesion. To achieving this, the authors loaded the mesopores of MSNs with two glycan-based structures: agarose for an antibacterial effect and heparin as an anticoagulant [202]. The agarose-heparin loaded MCM-41 nanoparticles were electrostatically immobilized onto the prepared device, i.e., an amino-modified silicone film. Therefore, the final coating presented a negative charge and exposed mesopores, which were able to produce effective agarose and heparin release. The system demonstrated a low hemolytic effect comparable with that of the previous model but with an additional antibacterial effect against *E. coli*, gaining additional applicability. However, regarding the design, the possibility of detaching SBA-15 particles within the bloodstream has to be considered as a potential risk and must be accounted for in the further development of such devices.

## 7. Challenges and Future Perspectives

As reviewed, the number of developing therapeutic hybrid nanosystems employing biomolecules together with mesoporous silica nanoparticles is enormous, as reported over the last few years. In general, and according to the literature here visited, cancer therapy is by far the most active research field and the one that exploits the most up-to-date technological advances such as PEGylation, controlled drug delivery, or the implementation of hybrid nanomaterials, among many others. However, such technology permitted developing alternative therapies to cancer, applied to other not-so-common diseases with a promising prognosis. This fact is largely due to the particular structure of MSNs, with a high capacity to load and release therapeutic molecules within their porous matrix. However, the advancement of knowledge and the need to adapt these nanomaterials to advanced preclinical in vivo models, with new aspects in loading and release systems, must be considered when designing these medical nanosystems. In this context, short therapeutic peptides and small molecules seem to be the ideal target for transfer to preclinical studies because they can be easily designed, developed, functionalized, and coupled. Among these new emerging therapeutic strategies with biomolecules that came to light recently, the most promising for implementation together with mesoporous silica materials seem to be immune therapy, gene modulation, and anti-infective nano-agents, as can be determined from the number and quality of related papers. In any case, there are different issues to be addressed regarding the nanocarriers themselves such as the absence of reliable in vivo pre-clinical studies, methods of clearance of the nanoplatforms, and how different particles properties (shape, size, pore symmetry and connectivity, and chemical composition) affect their application in nanomedicine.

## Figures and Tables

**Table 1:** Examples of therapeutic proteins delivered by silica-based nanocarriers.

Protein	Carrier Type	Protein Location	Loading Strategy	Cell Line(s)	In Vivo	Reference
***Anticancer proteins*** [52,53]						
Cytochrome C	MSNs	Mesopores	Pore filling	HeLa	None	[54,55]
	MSNs	Surface	Adsorption	None	None	[56]
	MSNs	Surface	Grafting	HeLa	None	[57]
	MSNs	Mesopores	Pore filling	SKOV3	None	[58]
Concanavalin A	MSNs	Surface	Grafting	MC3T3-E1, HOS	None	[59]
***Immunostimulating proteins and vaccines***						
IgG	HMSNs	Particle cavity	Cavity loading	HeLa	None	[60]
OVA	HMSNs	Mesopores	Pore filling	NIH3T3	Mice	[61,62]
OVA	DMOHS	Mesopores	Pore filling	None	Mice	[63]
CpG@OVA	MSNs	Mesopores	Pore filling	RAW264.7	Mice	[64]
CpG@OVA GM-CSF	MSNs@ MSRs	Mesopores Mesopores	Pore filling	None	Mice	[65]
Cyt c, IgG, Anti-pAkt	RSNs	Interparticles	In-pocket packing	None	None	[66]
IL-2	HMSNs	Particle cavity	Cavity loading	L929	Mice	[67]
ORF2	HMSNs	Surface	Adsorption	PK15	Mice	[68]
SWAP	MSNs	Surface	Adsorption	None	Mice	[69]
HSP700	MSNs	Surface	Adsorption	None	Mice	[70]
EspA	MSNs	Surface	Adsorption	None	Mice	[71]
rPb27	MSNs	Surface	Adsorption	HEK-293	Mice	[72]
***Enzymes***						
CA	MSNs	Mesopores	In-pore grafting	HeLa	None	[73]
CA or HPR	MSNs	Mesopores	In-pore grafting	None	None	[74]
β-Galactosidase	MSNs	Mesopores	Adsorption	N2a	None	[75]
SOD	MSNs	Surface	Grafting	HeLa	None	[76]
SOD or GPx	MSNs	Surface	Grafting	HeLa	None	[77]
Proteasomes	MSNs	Surface	Grafting	HEK-293, HeLa	None	[78]
***Growth factors***						
bFGF	MSNs	Mesopores	Microemulsion	HUVEC	None	[79]
BMP-2	MSNs	Surface	Adsorption	bMSCs	Mice	[80]
	MSN@SPION	Mesopores	Pore filling	bMSCs	None	[81]
***Antibacterial proteins***						
Lysozyme	MSNs	Surface	Grafting	*Escherichia coli*	Mice	[82]
	MSNs	Pores	Adsorption	*E. coli*	None	[83]
	HMSNs	Surface	Adsorption	*E. coli*	Mice	[84]
	HMSNs	Particle cavity	Cavity loading	*E. coli*	None	[85]
Concanavalin A	MSNs	Surface	Grafting	*E. coli*	None	[86]

Abbreviations: bFGF: basic fibroblast growth factor; BMP-2: bone morphogenetic protein 2; bMSCs: murine bone mesenchymal stem cells; CA: carbonic anhydrase; CpG@OVA: ovalbumin-loaded cytosine–phosphate–guanine (CpG) oligodeoxynucleotide; DMOHS: dendritic mesoporous organosilica hollow spheres; EspA: an immunogenic protein from enterohaemorrhagic *Escherichia Coli*; GM-CSF: murine granulocyte-macrophage colony-stimulating factor; GPx: glutathione peroxidase; HMSNs: hollow mesoporous silica nanoparticles; HRP: horseradish peroxidase; IgG: immunoglobulin G; IL-2: interleukin-2; ORF2: open reading frame from porcine circovirus type 2; OVA: chicken ovalbumin; RSNs: rough (non-porous and core–shell) silica nanoparticles; SOD: superoxide dismutase; SPIONs: superparamagnetic iron oxide nanoparticles.

**Table 2:** Examples of different types of therapeutic peptides delivered by mesoporous silica-based nanocarriers.

Peptide	Carrier	Pore Cargo	Release Mechanism	Cell Line(s)	In Vivo	Reference
***Anticancer Peptides***						
K_8_-Citraconate K_8_(RGD)_2_	MSNs	Doxorubicin	Electrostatic	COS7, U87 MG	None	[111]
TPP-K-(KLAKLAK)_2_-	MSNs	Topotecan	Electrostatic/Redox cleavage	KB	None	[112]
C-GRK_2_R_2_QR_3_P_2_Q-RGDS C-GKGG-D(KLAKLAK)_2_	MSNs	Doxorubicin	Redox cleavage	HeLa, COS7	None	[113]
(RGDWWW)_2_KC	MSNs	Doxorubicin	Redox cleavage	COS7, U87 MG	Mice	[114]
(KLAKLAK)_2_	MSNs	Doxorubicin	Enzymatic degradation Redox cleavage	HeLa	None	[115]
ε-poly-l-lysine C9h	MSNs	C9h	Enzymatic degradation Pore release	HeLa	None	[116]
RDG-Hylin a1	MSNs	RDG-Hylin a1	Pore release	HeLa Hep2	Mice	[117]
Pepstatin A	HMSNs	Pepstatin A	Release from cavity	MCF-7	None	[118]
NuBCP9	MSNs	NuBCP9	Pore release	HeLa, HEK293	Zebrafish	[119]
NuBCP9	PAMAM@MSNs	NuBCP9	Pore release upon PAMAM detachment	HepG2,H292, MCF-7,HeLa	Mice	[120]
***Immunostimulating Peptides***						
HGP100 TRP2	HMSNs	HGP100 TRP2	Lipid layer disassembly Release from cavity	BMDCs	None	[121]
***Antibacterial Peptides***						
LL37	SiO_2_/MSNs	None/LL37	Surface adsorption vs. pore release	*E. coli*	None	[122]
NZX	MSNs	NZX	Pore release	*Mycobacterium tuberculosis*	Mice	[123]
Melittin	MSNs@ MagMSNs	Melittin Ofloxacin	Pore release upon hyperthermia triggering	*Pseudomonas aeruginosa*	Mice	[124]
***Hormones and Growth Factors***						
Insulin	MSNs	cAMP	Glucose-mediated displacement	RIN-5F	None	[125]
	MSNs	Insulin	Glucose-sensitive polymer shell	None	None	[126]
	MSNs	Insulin	Pore release	Caco-2	None	[127]
Osteostatin	MSNs	Osteostatin	Pore release	MC3T3-E1	Rabbit	[128,129,130]
OGP	MSNs MS-HANs	OGP	Pore release	None	None	[131]
BMP-2	MSNs	Surface	None	BMSCs	Rat	[132]

For an illustrative revision of the coupling protocols employed for linking peptides onto nanocarriers, please check Reference [133]. Abbreviations: BMDCs: murine bone-marrow-derived dendritic cells; BMP-2: bone morphogenetic protein 2; BMSCs: rat bone mesenchymal stem cells; cAMP: cyclic adenosine monophosphate; MagMSNs: SPION@MSNs core–shell nanoparticles; MS-HANs: mesoporous silica–hydroxyapatite nanoparticles; OGP: osteogenic growth peptide; PAMAM: polyamidoamine dendrimer.

**Table 3:** Examples of different therapeutic nucleic acids delivered by silica-based nanocarriers.

Nucleic Acid	Carrier Type	NA Location	Loading Strategy	Cell Line(s)	In Vivo	Application	Reference
***DNA***							
pDNA	MSNs	Surface	Adsorption	Neuro-2A	None	Cancer	[146]
pDNA	MSNs	Surface	Adsorption	HEPA-1	None	Cancer	[147]
dsDNA	MSNs	Surface	Adsorption	HeLa	None	Cancer	[148]
dsDNA	MSNs	Surface	Adsorption	HL-60	None	Cancer	[149]
pDNA	IDMSMs	Surface	Adsorption	QGY-7703	Mice	Cancer	[150]
ssDNA	MSNs	Surface	Disulfide bond Maleimide	MCF-7	None	Cancer	[151]
dsDNA	HMSNs	Surface	Adsorption	MCF-7, A549, HepG2	None	Cancer	[152]
ssDNA	MSNs	Surface	Adsorption	MDA-MB-231	Mice	Cancer	[153]
ssDNA	MSNs	Surface	Adsorption	MCF-7, K-562, U2OS	Mice	Cancer	[154]
pDNA	MSNs	Surface	Adsorption	293T, RAW264.7	Mice	Hepatitis B	[155]
siRNA							
PKM2	MSNs	Surface	Adsorption	MDA-MB-231	Mice	Cancer	[156]
Cell-killing	MSNs	Mesopores	Adsorption	MDA-MB-231	None	Cancer	[157]
HER2	MSNs	Surface	Adsorption	BT474	None	Cancer	[158]
TGFβR-1	MSNs	Surface	Adsorption	RT3	Mice	Cancer	[159]
MDR1	MSNs	Surface	Adsorption	CAL27	None	Cancer	[160]
MDR1	MSNs	Surface	Adsorption	KBV	Mice	Cancer	[161]
Bcl-2	MSNs	Surface	Adsorption	SKOV-3, MCF-7	None	Cancer	[162]
Bcl-2	MSNs	Surface	Adsorption	SKOV-3	Mice	Cancer	[163]
TWIST	MSNs	Surface	Adsorption	F2, Ovcar8	Mice	Cancer	[164]
VEGF	MSNs	Surface	Adsorption	HeLa, HepG2	None	Cancer	[165]
VEGF	MSNs	Surface	Adsorption	HepG2, Huh7	None	Cancer	[166]
HSP47	MSNs	Surface	Adsorption	PMDF	Mice	Fibrosis	[167]
TnC	MSNs	Surface	Adsorption	rHSCs, mHSCs	None	Fibrosis	[168]
COLL1A1	MSNs	Surface	Adsorption	HDFs	Rat	Tissue regeneration	[169]
SOST	MSNs	Surface	Adsorption	MEF, HeLa	Mice	Osteoporosis	[170]
miRNA							
miR-155	MSNs	Mesopores	Adsorption	SW480	Mice	Cancer	[171]
miR-328	MSNs	Mesopores	Adsorption	SW480, SW620, HT-29, Lovo, Caco-2	Mice	Cancer	[172]
miR-34a miR-10b	MSNs	Mesopores	Adsorption	MDA-MB-231, MDA-MB-468	Mice	Cancer	[173]
miR-26a	MSNs	Mesopores	Adsorption	rBMSCs	None	Tissue regeneration	[174]
miR-200c siPlk1	MSNs	Mesopores	Adsorption	MDA-MB-231	Mice	Cancer	[175]

Abbreviations: IDMSN: imidazole dendritic mesoporous silica nanoparticles; PKM2: glycolytic enzyme pyruvate kinase; HER2: human epidermal growth factor receptor 2; TGFβR-1: transforming growth factor, beta receptor I; MDR1: multidrug resistance protein 1; Bcl-2: B-cell lymphoma 2; VEGF: vascular endothelial growth factor; HSP47: heat-shock protein 47; TnC: tenascin-C; COLL1A1: collagen type I; PMDFs: primary mouse dermal fibroblasts; HSCs: human stem cells; HDFs: human dermal fibroblasts; MEF: mouse embryonic fibroblastic cells; rBMSCs: rat bone marrow stromal cells.

## Abbreviations

ATP	Adenosine triphosphate
Bcl-2	B-cell lymphoma 2
bFGF	Basic fibroblast growth factor
BM	Biomacromolecule
BMP-2	Bone morphogenetic protein 2
bMSCs	Murine bone mesenchymal stem cells
BSA	Bovine serum albumin
CA	Carbonic anhydrase
cAMP	Cyclic adenosine monophosphate
COLL1A1	Collagen type 1
ConA	Concanavalin A
CS	Chondroitin sulfate
DMOHS	Dendritic mesoporous organosilica hollow spheres
DNA	Deoxyribonucleic acid
Cyt c	Cytochrome c
DOX	Doxorubicin
FA	Folic acid/folate
gG	Immunoglobulin G
GFP	Green fluorescent protein
GM-CSF	Murine granulocyte-macrophage colony-stimulating factor
GPx	Glutathione peroxidase
HMSNs	Hollow mesoporous silica nanoparticles
HA	Hyaluronic acid
HDFs	Human dermal fibroblasts
HOS	Human osteosarcoma
HRP	Horseradish peroxidase
HSCs	Human stem cells
HSP47	Heat-shock protein 47
IDMSN	Imidazole dendritic mesoporous silica nanoparticles
IL-2	Interleukin-2
kDa	Kilodaltons
LA	Lactobionic acid
LDL	Low-density lipoprotein
MCM-41	Mobil composition of matter formulation 41
MDR1	Multidrug resistance protein 1
MEF	Mouse embryonic fibroblastic cells
MPLA	Monophosphoryl lipid A
miRNA	Micro ribonucleic acid
mRNA	messenger ribonucleic acid
MSNs	Mesoporous silica nanoparticles
NA	Nucleic acid
NOX4	NADPH oxidase 4
OGP	Osteogenic growth peptide
OVA	Chicken ovalbumin
PAMAM	Polyamidoamine dendrimer
PDA	Polydopamine
PEG	Polyethylene glycol
PEI	Polyethyleneimine
pGFP	Fluorescent protein plasmid
PKM2	Glycolytic enzyme pyruvate kinase
PMDFs	Primary mouse dermal fibroblast
ROS	Oxygen species
RSNs	Rough (non-porous and core–shell) silica nanoparticles
SBA-15	Santa Barbara amorphous formulation 15
rBMSCs	Rat bone marrow stromal cells
RCA	Rolling circle amplification
shRNA	Short hairpin ribonucleic acid
siRNA	Small interfering ribonucleic acid
SO	Sorafenib
SOD	Superoxide dismutase
SPIONs	Superparamagnetic iron oxide nanoparticles.
TAT	Transactivator of transcription
TnC	Tenascin-C
TGFβR-1	Transforming growth factor beta receptor I
VEGF	Vascular endothelial growth factor

## References

[1] Vallet-Regi M., Rámila A., del Real R.P., Pérez-Pariente J. (2001). A New Property of MCM-41: Drug Delivery System. Chem. Mater..

[2] Yang J., Li L., Kopeček J. (2019). Biorecognition: A key to drug-free macromolecular therapeutics. Biomaterials.

[3] Sever R., Brugge J.S. (2015). Signal Transduction in Cancer. Cold Spring Harb. Perspect. Med..

[4] Núñez-Lozano R., Cano M., Pimentel B., de la Cueva-Méndez G. (2015). ‘Smartening’ anticancer therapeutic nanosystems using biomolecules. Curr. Opin. Biotechnol..

[5] Castillo R.R., Lozano D., González B., Manzano M., Izquierdo-Barba I., Vallet-Regí M. (2019). Advances in mesoporous silica nanoparticles for targeted stimuli-responsive drug delivery: An update. Expert Opin. Drug Deliv..

[6] Dempke W.C.M., Fenchel K., Uciechowski P., Dale S.P. (2017). Second-and third-generation drugs for immuno-oncology treatment—The more the better?. Eur. J. Cancer.

[7] Topalian S.L., Taube J.M., Anders R.A., Pardoll D.M. (2016). Mechanism-driven biomarkers to guide immune checkpoint blockade in cancer therapy. Nat. Rev. Cancer.

[8] Ribas A., Wolchok J.D. (2018). Cancer immunotherapy using checkpoint blockade. Science.

[9] Ahuja N., Sharma A.R., Baylin S.B. (2016). Epigenetic Therapeutics: A New Weapon in the War Against Cancer. Annu. Rev. Med..

[10] Das S.K., Menezes M.E., Bhatia S., Wang X.-Y., Emdad L., Sarkar D., Fisher P.B. (2015). Gene Therapies for Cancer: Strategies, Challenges and Successes. J. Cell. Physiol..

[11] Fukuhara H., Ino Y., Todo T. (2016). Oncolytic virus therapy: A new era of cancer treatment at dawn. Cancer Sci..

[12] Yamada H., Urata C., Aoyama Y., Osada S., Yamauchi Y., Kuroda K. (2012). Preparation of colloidal mesoporous silica nanoparticles with different diameters and their unique degradation behavior in static aqueous systems. Chem. Mater..

[13] Yamada H., Urata C., Ujiie H., Yamauchi Y., Kuroda K. (2013). Preparation of aqueous colloidal mesostructured and mesoporous silica nanoparticles with controlled particle size in a very wide range from 20 nm to 700 nm. Nanoscale.

[14] Knežević N.Ž., Durand J.-O. (2015). Large pore mesoporous silica nanomaterials for application in delivery of biomolecules. Nanoscale.

[15] Wu S.H., Lin H.P. (2013). Synthesis of mesoporous silica nanoparticles. Chem. Soc. Rev..

[16] Lindén M. (2018). Biodistribution and Excretion of Intravenously Injected Mesoporous Silica Nanoparticles: Implications for Drug Delivery Efficiency and Safety. The Enzymes.

[17] Croissant J.G., Fatieiev Y., Khashab N.M. (2017). Degradability and Clearance of Silicon, Organosilica, Silsesquioxane, Silica Mixed Oxide, and Mesoporous Silica Nanoparticles. Adv. Mater..

[18] Castillo R.R., Vallet-Regí M. (2019). Functional Mesoporous Silica Nanocomposites: Biomedical applications and Biosafety. Int. J. Mol. Sci..

[19] Castillo R.R., Colilla M., Vallet-Regí M. (2017). Advances in mesoporous silica-based nanocarriers for co-delivery and combination therapy against cancer. Expert Opin. Drug Deliv..

[20] Castillo R.R., Baeza A., Vallet-Regí M. (2017). Recent applications of the combination of mesoporous silica nanoparticles with nucleic acids: Development of bioresponsive devices, carriers and sensors. Biomater. Sci..

[21] Narayan R., Nayak U., Raichur A., Garg S. (2018). Mesoporous Silica Nanoparticles: A Comprehensive Review on Synthesis and Recent Advances. Pharmaceutics.

[22] Vallet-Regí M., Balas F., Colilla M., Manzano M. (2008). Bone-regenerative bioceramic implants with drug and protein controlled delivery capability. Prog. Solid State Chem..

[23] Anselmo A.C., Mitragotri S. (2015). A Review of Clinical Translation of Inorganic Nanoparticles. AAPS J..

[24] Anselmo A.C., Mitragotri S. (2019). Nanoparticles in the clinic: An update. Bioeng. Transl. Med..

[25] Florence A.T. (2018). Nanotechnologies for site specific drug delivery: Changing the narrative. Int. J. Pharm..

[26] Baeza A., Manzano M., Colilla M., Vallet-Regí M. (2016). Recent advances in mesoporous silica nanoparticles for antitumor therapy: Our contribution. Biomater. Sci..

[27] Singh R.K., Knowles J.C., Kim H.-W. (2019). Advances in nanoparticle development for improved therapeutics delivery: Nanoscale topographical aspect. J. Tissue Eng..

[28] Niu Y., Yu M., Meka A., Liu Y., Zhang J., Yang Y., Yu C. (2016). Understanding the contribution of surface roughness and hydrophobic modification of silica nanoparticles to enhanced therapeutic protein delivery. J. Mater. Chem. B..

[29] Song H., Yu M., Lu Y., Gu Z., Yang Y., Zhang M., Fu J., Yu C. (2017). Plasmid DNA Delivery: Nanotopography Matters. J. Am. Chem. Soc..

[30] Aznar E., Oroval M., Pascual L., Murguía J.R., Martínez-Máñez R., Sancenón F. (2016). Gated Materials for On-Command Release of Guest Molecules. Chem. Rev..

[31] Thi T.T.H., Nguyen T.N.Q., Hoang D.T., Nguyen D.H. (2019). Functionalized mesoporous silica nanoparticles and biomedical applications. Mater. Sci. Eng. C.

[32] Hu J.J., Xiao D., Zhang X.Z. (2016). Advances in Peptide Functionalization on Mesoporous Silica Nanoparticles for Controlled Drug Release. Small.

[33] Bruno B.J., Miller G.D., Lim C.S. (2013). Basics and recent advances in peptide and protein drug delivery. Ther. Deliv..

[34] Baker M. (2015). Reproducibility crisis: Blame it on the antibodies. Nature.

[35] Montenegro J.-M., Grazu V., Sukhanova A., Agarwal S., de la Fuente J.M., Nabiev I., Greiner A., Parak W.J. (2013). Controlled antibody/(bio-) conjugation of inorganic nanoparticles for targeted delivery. Adv. Drug Deliv. Rev..

[36] Cheng K., El-Boubbou K., Landry C.C. (2012). Binding of HIV-1 gp120 Glycoprotein to Silica Nanoparticles Modified with CD4 Glycoprotein and CD4 Peptide Fragments. ACS Appl. Mater. Interfaces.

[37] Kamegawa R., Naito M., Miyata K. (2018). Functionalization of silica nanoparticles for nucleic acid delivery. Nano Res..

[38] Benjaminsen R.V., Mattebjerg M.A., Henriksen J.R., Moghimi S.M., Andresen T.L. (2013). The Possible “Proton Sponge” Effect of Polyethylenimine (PEI) Does Not Include Change in Lysosomal pH. Mol. Ther..

[39] Boraschi D., Duschl A. (2014). Nanoparticles and the Immune System.

[40] Tu J., Boyle A.L., Friedrich H., Bomans P.H.H., Bussmann J., Sommerdijk N.A.J.M., Jiskoot W., Kros A. (2016). Mesoporous Silica Nanoparticles with Large Pores for the Encapsulation and Release of Proteins. ACS Appl. Mater. Interfaces.

[41] Kruk M. (2012). Access to Ultralarge-Pore Ordered Mesoporous Materials through Selection of Surfactant/Swelling-Agent Micellar Templates. Acc. Chem. Res..

[42] Gao Z., Zharov I. (2014). Large Pore Mesoporous Silica Nanoparticles by Templating with a Nonsurfactant Molecule, Tannic Acid. Chem. Mater..

[43] Balas F., Manzano M., Horcajada P., Vallet-Regi M. (2006). Confinement and controlled release of bisphosphonates on ordered mesoporous silica-based materials. J. Am. Chem. Soc..

[44] Wu M., Meng Q., Chen Y., Du Y., Zhang L., Li Y., Zhang L., Shi J. (2015). Large-Pore Ultrasmall Mesoporous Organosilica Nanoparticles: Micelle/Precursor Co-templating Assembly and Nuclear-Targeted Gene Delivery. Adv. Mater..

[45] Na H.-K., Kim M.-H., Park K., Ryoo S.-R., Lee K.E., Jeon H., Ryoo R., Hyeon C., Min D.-H. (2012). Efficient Functional Delivery of siRNA using Mesoporous Silica Nanoparticles with Ultralarge Pores. Small.

[46] Ashley C.E., Carnes E.C., Epler K.E., Padilla D.P., Phillips G.K., Castillo R.E., Wilkinson D.C., Wilkinson B.S., Burgard C.A., Kalinich R.M. (2012). Delivery of Small Interfering RNA by Peptide-Targeted Mesoporous Silica Nanoparticle-Supported Lipid Bilayers. ACS Nano.

[47] Akash M.S.H., Rehman K., Tariq M., Chen S. (2015). Development of therapeutic proteins: Advances and challenges. Turk. J. Biol..

[48] Liu H.-J., Xu P. (2019). Smart Mesoporous Silica Nanoparticles for Protein Delivery. Nanomaterials.

[49] Xu C., Lei C., Yu C. (2019). Mesoporous Silica Nanoparticles for Protein Protection and Delivery. Front. Chem..

[50] Yu M., Gu Z., Ottewell T., Yu C. (2017). Silica-based nanoparticles for therapeutic protein delivery. J. Mater. Chem. B.

[51] Deodhar G.V., Adams M.L., Trewyn B.G. (2017). Controlled release and intracellular protein delivery from mesoporous silica nanoparticles. Biotechnol. J..

[52] Karpiński T., Adamczak A. (2018). Anticancer Activity of Bacterial Proteins and Peptides. Pharmaceutics.

[53] Liu X., Wu F., Ji Y., Yin L. (2019). Recent Advances in Anti-cancer Protein/Peptide Delivery. Bioconjug. Chem..

[54] Slowing I.I., Trewyn B.G., Lin V.S.-Y. (2007). Mesoporous Silica Nanoparticles for Intracellular Delivery of Membrane-Impermeable Proteins. J. Am. Chem. Soc..

[55] Méndez J., Morales Cruz M., Delgado Y., Figueroa C.M., Orellano E.A., Morales M., Monteagudo A., Griebenow K. (2014). Delivery of chemically glycosylated cytochrome c immobilized in mesoporous silica nanoparticles induces apoptosis in HeLa cancer cells. Mol. Pharm..

[56] Shang W., Nuffer J.H., Muñiz-Papandrea V.A., Colón W., Siegel R.W., Dordick J.S. (2009). Cytochrome c on silica nanoparticles: Influence of nanoparticle size on protein structure, stability, and activity. Small.

[57] Huang W.-Y., Davies G.-L., Davis J.J. (2013). Engineering Cytochrome-Modified Silica Nanoparticles To Induce Programmed Cell Death. Chem. A Eur. J..

[58] Choi E., Lim D.-K., Kim S. (2020). Hydrolytic surface erosion of mesoporous silica nanoparticles for efficient intracellular delivery of cytochrome c. J. Colloid Interface Sci..

[59] Martínez-Carmona M., Lozano D., Colilla M., Vallet-Regí M. (2018). Lectin-conjugated pH-responsive mesoporous silica nanoparticles for targeted bone cancer treatment. Acta Biomater..

[60] Lim J.-S., Lee K., Choi J.-N., Hwang Y.-K., Yun M.-Y., Kim H.-J., Won Y.S., Kim S.-J., Kwon H., Huh S. (2012). Intracellular protein delivery by hollow mesoporous silica capsules with a large surface hole. Nanotechnology.

[61] Wang X., Li X., Ito A., Yoshiyuki K., Sogo Y., Watanabe Y., Yamazaki A., Ohno T., Tsuji N.M. (2016). Silica Nanospheres: Hollow Structure Improved Anti-Cancer Immunity of Mesoporous Silica Nanospheres In Vivo. Small.

[62] Wang X., Li X., Yoshiyuki K., Watanabe Y., Sogo Y., Ohno T., Tsuji N.M., Ito A. (2016). Comprehensive Mechanism Analysis of Mesoporous-Silica-Nanoparticle-Induced Cancer Immunotherapy. Adv. Healthc. Mater..

[63] Yang Y., Lu Y., Abbaraju P.L., Zhang J., Zhang M., Xiang G., Yu C. (2017). Multi-shelled Dendritic Mesoporous Organosilica Hollow Spheres: Roles of Composition and Architecture in Cancer Immunotherapy. Angew. Chem. Int. Ed..

[64] Cha B.G., Jeong J.H., Kim J. (2018). Extra-Large Pore Mesoporous Silica Nanoparticles Enabling Co-Delivery of High Amounts of Protein Antigen and Toll-like Receptor 9 Agonist for Enhanced Cancer Vaccine Efficacy. ACS Cent. Sci..

[65] Nguyen T.L., Cha B.G., Choi Y., Im J., Kim J. (2020). Injectable dual-scale mesoporous silica cancer vaccine enabling efficient delivery of antigen/adjuvant-loaded nanoparticles to dendritic cells recruited in local macroporous scaffold. Biomaterials.

[66] Niu Y., Yu M., Zhang J., Yang Y., Xu C., Yeh M., Taran E., Hou J.J.C., Gray P.P., Yu C. (2015). Synthesis of silica nanoparticles with controllable surface roughness for therapeutic protein delivery. J. Mater. Chem. B.

[67] Kong M., Tang J., Qiao Q., Wu T., Qi Y., Tan S., Gao X., Zhang Z. (2017). Biodegradable Hollow Mesoporous Silica Nanoparticles for Regulating Tumor Microenvironment and Enhancing Antitumor Efficiency. Theranostics.

[68] Guo H.C., Feng X.M., Sun S.Q., Wei Y.Q., Sun D.H., Liu X.T., Liu Z.X., Luo J.X., Yin H. (2012). Immunization of mice by hollow mesoporous silica nanoparticles as carriers of porcine circovirus type 2 ORF2 protein. Virol. J..

[69] Oliveira D.C.d.P., de Barros A.L.B., Belardi R.M., de Goes A.M., de Oliveira Souza B.K., Soares D.C.F. (2016). Mesoporous silica nanoparticles as a potential vaccine adjuvant against Schistosoma mansoni. J. Drug Deliv. Sci. Technol..

[70] Virginio V.G., Bandeira N.C., dos Anjos Leal F.M., Lancellotti M., Zaha A., Ferreira H.B. (2017). Assessment of the adjuvant activity of mesoporous silica nanoparticles in recombinant Mycoplasma hyopneumoniae antigen vaccines. Heliyon.

[71] Hajizade A., Salmanian A.H., Amani J., Ebrahimi F., Arpanaei A. (2018). EspA-loaded mesoporous silica nanoparticles can efficiently protect animal model against enterohaemorrhagic E. coli O157: H7. Artif. Cells Nanomed. Biotechnol..

[72] Ferreira Soares D.C., Soares L.M., Miranda de Goes A., Melo E.M., Branco de Barros A.L., Alves Santos Bicalho T.C., Leao N.M., Tebaldi M.L. (2020). Mesoporous SBA-16 silica nanoparticles as a potential vaccine adjuvant against Paracoccidioides brasiliensis. Microporous Mesoporous Mater..

[73] Méndez J., Monteagudo A., Griebenow K. (2012). Stimulus-responsive controlled release system by covalent immobilization of an enzyme into mesoporous silica nanoparticles. Bioconjug. Chem..

[74] Gößl D., Singer H., Chiu H.Y., Schmidt A., Lichtnecker M., Engelke H., Bein T. (2019). Highly active enzymes immobilized in large pore colloidal mesoporous silica nanoparticles. N. J. Chem..

[75] Xu C., Yu M., Noonan O., Zhang J., Song H., Zhang H., Lei C., Niu Y., Huang X., Yang Y. (2015). Core-Cone Structured Monodispersed Mesoporous Silica Nanoparticles with Ultra-large Cavity for Protein Delivery. Small.

[76] Chen Y.P., Chen C.T., Hung Y., Chou C.M., Liu T.P., Liang M.R., Chen C.T., Mou C.Y. (2013). A new strategy for intracellular delivery of enzyme using mesoporous silica nanoparticles: Superoxide dismutase. J. Am. Chem. Soc..

[77] Lin Y.H., Chen Y.P., Liu T.P., Chien F.C., Chou C.M., Chen C.T., Mou C.Y. (2016). Approach to Deliver Two Antioxidant Enzymes with Mesoporous Silica Nanoparticles into Cells. ACS Appl. Mater. Interfaces.

[78] Han D.H., Na H.-K., Choi W.H., Lee J.H., Kim Y.K., Won C., Lee S.-H., Kim K.P., Kuret J., Min D.-H. (2014). Direct cellular delivery of human proteasomes to delay tau aggregation. Nat. Commun..

[79] Zhang J., Postovit L.M., Wang D., Gardiner R.B., Harris R., Abdul M.M., Thomas A.A. (2009). In situ loading of basic fibroblast growth factor within porous silica nanoparticles for a prolonged release. Nanoscale Res. Lett..

[80] Gan Q., Zhu J., Yuan Y., Liu H., Qian J., Li Y., Liu C. (2015). A dual-delivery system of pH-responsive chitosan-functionalized mesoporous silica nanoparticles bearing BMP-2 and dexamethasone for enhanced bone regeneration. J. Mater. Chem. B.

[81] Gan Q., Zhu J., Yuan Y., Liu C. (2016). pH-Responsive Fe3O4 Nanopartilces-Capped Mesoporous Silica Supports for Protein Delivery. J. Nanosci. Nanotechnol..

[82] Li L.-L., Wang H. (2013). Enzyme-coated mesoporous silica nanoparticles as efficient antibacterial agents in vivo. Adv. Healthc. Mater..

[83] Wang Y., Nor Y.A., Song H., Yang Y., Xu C., Yu M., Yu C. (2016). Small-sized and large-pore dendritic mesoporous silica nanoparticles enhance antimicrobial enzyme delivery. J. Mater. Chem. B.

[84] Song H., Ahmad Nor Y., Yu M., Yang Y., Zhang J., Zhang H., Xu C., Mitter N., Yu C. (2016). Silica Nanopollens Enhance Adhesion for Long-Term Bacterial Inhibition. J. Am. Chem. Soc..

[85] Xu C., He Y., Li Z., Ahmad Nor Y., Ye Q. (2018). Nanoengineered hollow mesoporous silica nanoparticles for the delivery of antimicrobial proteins into biofilms. J. Mater. Chem. B.

[86] Martínez-Carmona M., Izquierdo-Barba I., Colilla M., Vallet-Regí M. (2019). Concanavalin A-targeted mesoporous silica nanoparticles for infection treatment. Acta Biomater..

[87] Wang X., Li X., Ito A., Sogo Y., Ohno T. (2013). Particle-size-dependent toxicity and immunogenic activity of mesoporous silica-based adjuvants for tumor immunotherapy. Acta Biomater..

[88] Li X., Wang X., Sogo Y., Ohno T., Onuma K., Ito A. (2013). Mesoporous Silica-Calcium Phosphate-Tuberculin Purified Protein Derivative Composites as an Effective Adjuvant for Cancer Immunotherapy. Adv. Healthc. Mater..

[89] Wang X., Li X., Ito A., Watanabe Y., Sogo Y., Tsuji N.M., Ohno T. (2016). Stimulation of In Vivo Antitumor Immunity with Hollow Mesoporous Silica Nanospheres. Angew. Chem. Int. Ed..

[90] Kim M.-G., Park J.Y., Shon Y., Kim G., Shim G., Oh Y.-K. (2014). Nanotechnology and vaccine development. Asian J. Pharm. Sci..

[91] Pati R., Shevtsov M., Sonawane A. (2018). Nanoparticle Vaccines Against Infectious Diseases. Front. Immunol..

[92] Villegas M.R., Baeza A., Vallet-Regí M. (2018). Nanotechnological Strategies for Protein Delivery. Molecules.

[93] Zhao M., Liu Y., Hsieh R.S., Wang N., Tai W., Joo K.-I., Wang P., Gu Z., Tang Y. (2014). Clickable Protein Nanocapsules for Targeted Delivery of Recombinant p53 Protein. J. Am. Chem. Soc..

[94] Villegas M.R., Baeza A., Usategui A., Ortiz-Romero P.L., Pablos J.L., Vallet-Regí M. (2018). Collagenase nanocapsules: An approach to fibrosis treatment. Acta Biomater..

[95] Mohammed G.K., Obaidat R.M., Assaf S., Khanfar M., Al-Taani B. (2017). Formulations and Technologies in Growth Hormone Delivery. Int. J. Pharm. Pharm. Sci..

[96] Rohrer T.R., Horikawa R., Kappelgaard A.-M. (2017). Growth hormone delivery devices: Current features and potential for enhanced treatment adherence. Expert Opin. Drug Deliv..

[97] Kuijpers A.J., Engbers G.H., van Wachem P.B., Krijgsveld J., Zaat S.A., Dankert J., Feijen J. (1998). Controlled delivery of antibacterial proteins from biodegradable matrices. J. Control. Release.

[98] Jenssen H., Hancock R.E.W. (2009). Antimicrobial properties of lactoferrin. Biochimie.

[99] Zhu Y., Feijen J., Zhong Z. (2018). Dual-targeted nanomedicines for enhanced tumor treatment. Nano Today.

[100] Song Y., Du D., Li L., Xu J., Dutta P., Lin Y. (2017). In Vitro Study of Receptor-Mediated Silica Nanoparticles Delivery across Blood–Brain Barrier. ACS Appl. Mater. Interfaces.

[101] Kalmouni M., Al-Hosani S., Magzoub M. (2019). Cancer targeting peptides. Cell. Mol. Life Sci..

[102] Zhao N., Qin Y., Liu H., Cheng Z. (2018). Tumor-Targeting Peptides: Ligands for Molecular Imaging and Therapy. Anticancer Agents Med. Chem..

[103] Robinson J.A. (2019). Folded Synthetic Peptides and Other Molecules Targeting Outer Membrane Protein Complexes in Gram-Negative Bacteria. Front. Chem..

[104] Malanovic N., Lohner K. (2016). Antimicrobial Peptides Targeting Gram-Positive Bacteria. Pharmaceuticals.

[105] Jiang Z., Guan J., Qian J., Zhan C. (2019). Peptide ligand-mediated targeted drug delivery of nanomedicines. Biomater. Sci..

[106] Marqus S., Pirogova E., Piva T.J. (2017). Evaluation of the use of therapeutic peptides for cancer treatment. J. Biomed. Sci..

[107] Gaspar D., Salomé Veiga A., Castanho M.A.R.B. (2013). From antimicrobial to anticancer peptides. A review. Front. Microbiol..

[108] Kurrikoff K., Aphkhazava D., Langel Ü. (2019). The future of peptides in cancer treatment. Curr. Opin. Pharmacol..

[109] Mahlapuu M., Håkansson J., Ringstad L., Björn C. (2016). Antimicrobial Peptides: An Emerging Category of Therapeutic Agents. Front. Cell Infect. Microbiol..

[110] Lau J.L., Dunn M.K. (2018). Therapeutic peptides: Historical perspectives, current development trends, and future directions. Bioorg. Med. Chem..

[111] Luo G.-F., Chen W.-H., Liu Y., Zhang J., Cheng S.-X., Zhuo R.-X., Zhang X.-Z. (2013). Charge-reversal plug gate nanovalves on peptide-functionalized mesoporous silica nanoparticles for targeted drug delivery. J. Mater. Chem. B.

[112] Luo G.F., Chen W.H., Liu Y., Lei Q., Zhuo R.X., Zhang X.Z. (2014). Multifunctional enveloped mesoporous silica nanoparticles for subcellular co-delivery of drug and therapeutic peptide. Sci. Rep..

[113] Cheng Y.J., Zeng X., Cheng D.B., Xu X.D., Zhang X.Z., Zhuo R.X., He F. (2016). Functional mesoporous silica nanoparticles (MSNs) for highly controllable drug release and synergistic therapy. Colloids Surfaces B Biointerfaces.

[114] Xiao D., Hu J.-J., Zhu J.-Y., Wang S.-B., Zhuo R.-X., Zhang X.-Z. (2016). A redox-responsive mesoporous silica nanoparticle with a therapeutic peptide shell for tumor targeting synergistic therapy. Nanoscale.

[115] Zhang J., Shen B., Chen L., Chen L., Meng Y., Feng J. (2019). A dual-sensitive mesoporous silica nanoparticle based drug carrier for cancer synergetic therapy. Colloids Surfaces B Biointerfaces.

[116] De la Torre C., Domínguez-Berrocal L., Murguía J.R., Marcos M.D., Martínez-Máñez R., Bravo J., Sancenón F. (2018). ϵ-Polylysine-Capped Mesoporous Silica Nanoparticles as Carrier of the C9h Peptide to Induce Apoptosis in Cancer Cells. Chem. A Eur. J..

[117] Cao J., Zhang Y., Shan Y., Wang J., Liu F., Liu H., Xing G., Lei J., Zhou J. (2017). A pH-dependent Antibacterial Peptide Release Nano-system Blocks Tumor Growth in vivo without Toxicity. Sci. Rep..

[118] Rahmani S., Budimir J., Sejalon M., Daurat M., Aggad D., Vivès E., Raehm L., Garcia M., Lichon L., Gary-Bobo M. (2019). Large pore mesoporous silica and organosilica nanoparticles for pepstatin A delivery in breast cancer cells. Molecules.

[119] Wu Y., Ge P., Xu W., Li M., Kang Q., Zhang X., Xie J. (2020). Cancer-targeted and intracellular delivery of Bcl-2-converting peptide with functional macroporous silica nanoparticles for biosafe treatment. Mater. Sci. Eng. C.

[120] Xie J., Xu W., Wu Y., Niu B., Zhang X. (2020). Macroporous organosilicon nanocomposites co-deliver Bcl2-converting peptide and chemotherapeutic agent for synergistic treatment against multidrug resistant cancer. Cancer Lett..

[121] Xie J., Yang C., Liu Q., Li J., Liang R., Shen C., Zhang Y., Wang K., Liu L., Shezad K. (2017). Encapsulation of Hydrophilic and Hydrophobic Peptides into Hollow Mesoporous Silica Nanoparticles for Enhancement of Antitumor Immune Response. Small.

[122] Braun K., Pochert A., Lindén M., Davoudi M., Schmidtchen A., Nordström R., Malmsten M. (2016). Membrane interactions of mesoporous silica nanoparticles as carriers of antimicrobial peptides. J. Colloid Interface Sci..

[123] Tenland E., Pochert A., Krishnan N., Rao K.U., Kalsum S., Braun K., Glegola-Madejska I., Lerm M., Robertson B.D., Lindén M. (2019). Effective delivery of the anti-mycobacterial peptide NZX in mesoporous silica nanoparticles. PLoS ONE.

[124] Yu Q., Deng T., Lin F.-C., Zhang B., Zink J.I. (2020). Supramolecular Assemblies of Heterogeneous Mesoporous Silica Nanoparticles to Co-deliver Antimicrobial Peptides and Antibiotics for Synergistic Eradication of Pathogenic Biofilms. ACS Nano.

[125] Zhao Y., Trewyn B.G., Slowing I.I., Lin V.S.-Y. (2009). Mesoporous Silica Nanoparticle-Based Double Drug Delivery System for Glucose-Responsive Controlled Release of Insulin and Cyclic AMP. J. Am. Chem. Soc..

[126] Sun L., Zhang X., Zheng C., Wu Z., Li C. (2013). A pH gated, glucose-sensitive nanoparticle based on worm-like mesoporous silica for controlled insulin release. J. Phys. Chem. B.

[127] Zakeri Siavashani A., Haghbin Nazarpak M., Fayyazbakhsh F., Toliyat T., McInnes S.J.P., Solati-Hashjin M. (2016). Effect of amino-functionalization on insulin delivery and cell viability for two types of silica mesoporous structures. J. Mater. Sci..

[128] Lozano D., Manzano M., Doadrio J.C., Salinas A.J., Vallet-Regí M., Gómez-Barrena E., Esbrit P. (2010). Osteostatin-loaded bioceramics stimulate osteoblastic growth and differentiation. Acta Biomater..

[129] Trejo C.G., Lozano D., Manzano M., Doadrio J.C., Salinas A.J., Dapía S., Gómez-Barrena E., Vallet-Regí M., García-Honduvilla N., Buján J. (2010). The osteoinductive properties of mesoporous silicate coated with osteostatin in a rabbit femur cavity defect model. Biomaterials.

[130] Lozano D., Trejo C.G., Gómez-Barrena E., Manzano M., Doadrio J.C., Salinas A.J., Vallet-Regí M., García-Honduvilla N., Esbrit P., Buján J. (2012). Osteostatin-loaded onto mesoporous ceramics improves the early phase of bone regeneration in a rabbit osteopenia model. Acta Biomater..

[131] Mendes L.S., Saska S., Martines M.A.U., Marchetto R. (2013). Nanostructured materials based on mesoporous silica and mesoporous silica/apatite as osteogenic growth peptide carriers. Mater. Sci. Eng. C.

[132] Zhou X., Feng W., Qiu K., Chen L., Wang W., Nie W., Mo X., He C. (2015). BMP-2 Derived Peptide and Dexamethasone Incorporated Mesoporous Silica Nanoparticles for Enhanced Osteogenic Differentiation of Bone Mesenchymal Stem Cells. ACS Appl. Mater. Interfaces.

[133] Du A.W., Stenzel M.H. (2014). Drug carriers for the delivery of therapeutic peptides. Biomacromolecules.

[134] Mader J.S., Hoskin D.W. (2006). Cationic antimicrobial peptides as novel cytotoxic agents for cancer treatment. Expert Opin. Investig. Drugs.

[135] Torchilin V.P. (2008). Tat peptide-mediated intracellular delivery of pharmaceutical nanocarriers. Adv. Drug Deliv. Rev..

[136] Boohaker R.J., Lee M.W., Vishnubhotla P., Perez J.L.M., Khaled A.R. (2012). The Use of Therapeutic Peptides to Target and to Kill Cancer Cells. Curr. Med. Chem..

[137] Naz S., Wang M., Han Y., Hu B., Teng L., Zhou J., Zhang H., Chen J. (2019). Enzyme-responsive mesoporous silica nanoparticles for tumor cells and mitochondria multistage-targeted drug delivery. Int. J. Nanomed..

[138] Kolluri S.K., Zhu X., Zhou X., Lin B., Chen Y., Sun K., Tian X., Town J., Cao X., Lin F. (2008). A Short Nur77-Derived Peptide Converts Bcl-2 from a Protector to a Killer. Cancer Cell.

[139] Nordström R., Malmsten M. (2017). Delivery systems for antimicrobial peptides. Adv. Colloid Interface Sci..

[140] Braun K., Pochert A., Gerber M., Raber H.F., Lindén M. (2017). Influence of mesopore size and peptide aggregation on the adsorption and release of a model antimicrobial peptide onto/from mesoporous silica nanoparticles in vitro. Mol. Syst. Des. Eng..

[141] Tenland E., Krishnan N., Rönnholm A., Kalsum S., Puthia M., Mörgelin M., Davoudi M., Otrocka M., Alaridah N., Glegola-Madejska I. (2018). A novel derivative of the fungal antimicrobial peptide plectasin is active against Mycobacterium tuberculosis. Tuberculosis.

[142] Heras C., Sanchez-Salcedo S., Lozano D., Peña J., Esbrit P., Vallet-Regi M., Salinas A.J. (2019). Osteostatin potentiates the bioactivity of mesoporous glass scaffolds containing Zn 2+ ions in human mesenchymal stem cells. Acta Biomater..

[143] Pérez R., Sanchez-Salcedo S., Lozano D., Heras C., Esbrit P., Vallet-Regí M., Salinas A.J. (2018). Osteogenic effect of ZnO-mesoporous glasses loaded with osteostatin. Nanomaterials.

[144] Nayerossadat N., Ali P., Maedeh T. (2012). Viral and nonviral delivery systems for gene delivery. Adv. Biomed. Res..

[145] Cha W., Fan R., Miao Y., Zhou Y., Qin C., Shan X., Wan X., Li J. (2017). Mesoporous Silica Nanoparticles as Carriers for Intracellular Delivery of Nucleic Acids and Subsequent Therapeutic Applications. Molecules.

[146] Zarei H., Kazemi Oskuee R., Hanafi-Bojd M.Y., Gholami L., Ansari L., Malaekeh-Nikouei B. (2019). Enhanced gene delivery by polyethyleneimine coated mesoporous silica nanoparticles. Pharm. Dev. Technol..

[147] Xia T., Kovochich M., Liong M., Meng H., Kabehie S., George S., Zink J.I., Nel A.E. (2009). Polyethyleneimine Coating Enhances the Cellular Uptake of Mesoporous Silica Nanoparticles and Allows Safe Delivery of siRNA and DNA Constructs. ACS Nano.

[148] Wang Y., Shang X., Liu J., Guo Y. (2018). ATP mediated rolling circle amplification and opening DNA-gate for drug delivery to cell. Talanta.

[149] Wang S., Liu F., Li X.-L. (2017). Monitoring of “on-demand” drug release using dual tumor marker mediated DNA-capped versatile mesoporous silica nanoparticles. Chem. Commun..

[150] Li Z., Zhang L., Tang C., Yin C. (2017). Co-Delivery of Doxorubicin and Survivin shRNA-Expressing Plasmid Via Microenvironment-Responsive Dendritic Mesoporous Silica Nanoparticles for Synergistic Cancer Therapy. Pharm. Res..

[151] Sun P., Leidner A., Weigel S., Weidler P.G., Heissler S., Scharnweber T., Niemeyer C.M. (2019). Biopebble Containers: DNA-Directed Surface Assembly of Mesoporous Silica Nanoparticles for Cell Studies. Small.

[152] Li Y., Chen Y., Pan W., Yu Z., Yang L., Wang H., Li N., Tang B. (2017). Nanocarriers with multi-locked DNA valves targeting intracellular tumor-related mRNAs for controlled drug release. Nanoscale.

[153] Pascual L., Cerqueira-Coutinho C., García-Fernández A., de Luis B., Bernardes E.S., Albernaz M.S., Missailidis S., Martínez-Máñez R., Santos-Oliveira R., Orzaez M. (2017). MUC1 aptamer-capped mesoporous silica nanoparticles for controlled drug delivery and radio-imaging applications. Nanomed. Nanotechnol. Biol. Med..

[154] Srivastava P., Hira S.K., Sharma A., Kashif M., Srivastava P., Srivastava D.N., Singh R.A., Manna P.P. (2018). Telomerase Responsive Delivery of Doxorubicin from Mesoporous Silica Nanoparticles in Multiple Malignancies: Therapeutic Efficacies against Experimental Aggressive Murine Lymphoma. Bioconjug. Chem..

[155] Wang J., Zhu R., Gao B., Wu B., Li K., Sun X., Liu H., Wang S. (2014). The enhanced immune response of hepatitis B virus DNA vaccine using SiO2@LDH nanoparticles as an adjuvant. Biomaterials.

[156] Shen J., Kim H.-C., Su H., Wang F., Wolfram J., Kirui D., Mai J., Mu C., Ji L.-N., Mao Z.-W. (2014). Cyclodextrin and Polyethylenimine Functionalized Mesoporous Silica Nanoparticles for Delivery of siRNA Cancer Therapeutics. Theranostics.

[157] Prabhakar N., Zhang J., Desai D., Casals E., Gulin-Sarfraz T., Näreoja T., Westermarck J., Rosenholm J. (2016). Stimuli-responsive hybrid nanocarriers developed by controllable integration of hyperbranched PEI with mesoporous silica nanoparticles for sustained intracellular siRNA delivery. Int. J. Nanomed..

[158] Ngamcherdtrakul W., Sangvanich T., Reda M., Gu S., Bejan D., Yantasee W. (2018). Lyophilization and stability of antibody-conjugated mesoporous silica nanoparticle with cationic polymer and PEG for siRNA delivery. Int. J. Nanomed..

[159] Lio D.C.S., Liu C., Oo M.M.S., Wiraja C., Teo M.H.Y., Zheng M., Chew S.W.T., Wang X., Xu C. (2019). Transdermal delivery of small interfering RNAs with topically applied mesoporous silica nanoparticles for facile skin cancer treatment. Nanoscale.

[160] Wang D., Xu X., Zhang K., Sun B., Wang L., Meng L., Liu Q., Zheng C., Yang B., Sun H. (2017). Codelivery of doxorubicin and MDR1-siRNA by mesoporous silica nanoparticles-polymerpolyethylenimine to improve oral squamous carcinoma treatment. Int. J. Nanomed..

[161] Shi X.-L., Li Y., Zhao L.-M., Su L.-W., Ding G. (2019). Delivery of MTH1 inhibitor (TH287) and MDR1 siRNA via hyaluronic acid-based mesoporous silica nanoparticles for oral cancers treatment. Colloids Surfaces B Biointerfaces.

[162] Pan Q.-S., Chen T.-T., Nie C.-P., Yi J.-T., Liu C., Hu Y.-L., Chu X. (2018). In Situ Synthesis of Ultrathin ZIF-8 Film-Coated MSNs for Codelivering Bcl 2 siRNA and Doxorubicin to Enhance Chemotherapeutic Efficacy in Drug-Resistant Cancer Cells. ACS Appl. Mater. Interfaces.

[163] Choi E., Lee J., Kwon I.C., Lim D.-K., Kim S. (2019). Cumulative directional calcium gluing between phosphate and silicate: A facile, robust and biocompatible strategy for siRNA delivery by amine-free non-positive vector. Biomaterials.

[164] Shahin S.A., Wang R., Simargi S.I., Contreras A., Parra Echavarria L., Qu L., Wen W., Dellinger T., Unternaehrer J., Tamanoi F. (2018). Hyaluronic acid conjugated nanoparticle delivery of siRNA against TWIST reduces tumor burden and enhances sensitivity to cisplatin in ovarian cancer. Nanomed. Nanotechnol. Biol. Med..

[165] Zheng G., Shen Y., Zhao R., Chen F., Zhang Y., Xu A., Shao J. (2017). Dual-Targeting Multifuntional Mesoporous Silica Nanocarrier for Codelivery of siRNA and Ursolic Acid to Folate Receptor Overexpressing Cancer Cells. J. Agric. Food Chem..

[166] Zheng G., Zhao R., Xu A., Shen Z., Chen X., Shao J. (2018). Co-delivery of sorafenib and siVEGF based on mesoporous silica nanoparticles for ASGPR mediated targeted HCC therapy. Eur. J. Pharm. Sci..

[167] Morry J., Ngamcherdtrakul W., Gu S., Goodyear S.M., Castro D.J., Reda M.M., Sangvanich T., Yantasee W. (2015). Dermal delivery of HSP47 siRNA with NOX4-modulating mesoporous silica-based nanoparticles for treating fibrosis. Biomaterials.

[168] Vivero-Escoto J.L., Vadarevu H., Juneja R., Schrum L.W., Benbow J.H. (2019). Nanoparticle mediated silencing of tenascin C in hepatic stellate cells: Effect on inflammatory gene expression and cell migration. J. Mater. Chem. B.

[169] Pinese C., Lin J., Milbreta U., Li M., Wang Y., Leong K.W., Chew S.Y. (2018). Sustained delivery of siRNA/mesoporous silica nanoparticle complexes from nanofiber scaffolds for long-term gene silencing. Acta Biomater..

[170] Mora-Raimundo P., Lozano D., Manzano M., Vallet-Regí M. (2019). Nanoparticles to Knockdown Osteoporosis-Related Gene and Promote Osteogenic Marker Expression for Osteoporosis Treatment. ACS Nano.

[171] Li Y., Duo Y., Bi J., Zeng X., Mei L., Bao S., He L., Shan A., Zhang Y., Yu X. (2018). Targeted delivery of anti-miR-155 by functionalized mesoporous silica nanoparticles for colorectal cancer therapy. Int. J. Nanomed..

[172] Li Y., Duo Y., Zhai P., He L., Zhong K., Zhang Y., Huang K., Luo J., Zhang H., Yu X. (2018). Dual targeting delivery of miR-328 by functionalized mesoporous silica nanoparticles for colorectal cancer therapy. Nanomedicine.

[173] Ahir M., Upadhyay P., Ghosh A., Sarker S., Bhattacharya S., Gupta P., Ghosh S., Chattopadhyay S., Adhikary A. (2020). Delivery of dual miRNA through CD44-targeted mesoporous silica nanoparticles for enhanced and effective triple-negative breast cancer therapy. Biomater. Sci..

[174] Yan J., Lu X., Zhu X., Hu X., Wang L., Qian J., Zhang F., Liu M. (2020). Effects of miR-26a on Osteogenic Differentiation of Bone Marrow Mesenchymal Stem Cells by a Mesoporous Silica Nanoparticle-PEI-Peptide System. Int. J. Nanomed..

[175] Wang Y., Xie Y., Kilchrist K.V., Li J., Duvall C.L., Oupický D. (2020). Endosomolytic and Tumor-Penetrating Mesoporous Silica Nanoparticles for siRNA/miRNA Combination Cancer Therapy. ACS Appl. Mater. Interfaces.

[176] Maimaitiyiming Y., Hong D.F., Yang C., Naranmandura H. (2019). Novel insights into the role of aptamers in the fight against cancer. J. Cancer Res. Clin. Oncol..

[177] Ingolotti M., Kawalekar O., Shedlock D.J., Muthumani K., Weiner D.B. (2010). DNA vaccines for targeting bacterial infections. Expert Rev. Vaccines.

[178] Darvishi B., Farahmand L., Majidzadeh-A K. (2017). Stimuli-Responsive Mesoporous Silica NPs as Non-viral Dual siRNA/Chemotherapy Carriers for Triple Negative Breast Cancer. Mol. Ther. Nucleic Acids.

[179] Lopes C.F.B., de Angelis B.B., Prudente H.M., de Souza B.V.G., Cardoso S.V., de Azambuja Ribeiro R.I.M. (2012). Concomitant consumption of marijuana, alcohol and tobacco in oral squamous cell carcinoma development and progression: Recent advances and challenges. Arch. Oral Biol..

[180] Nishida N., Yano H., Nishida T., Kamura T., Kojiro M. (2006). Angiogenesis in cancer. Vasc. Health Risk Manag..

[181] Richter K., Konzack A., Pihlajaniemi T., Heljasvaara R., Kietzmann T. (2015). Redox-fibrosis: Impact of TGFβ1 on ROS generators, mediators and functional consequences. Redox Biol..

[182] Mora-Raimundo P., Manzano M., Vallet-Regí M. (2017). Nanoparticles for the treatment of osteoporosis. AIMS Bioeng..

[183] Lozano D., Sánchez-Salcedo S., Portal-Núñez S., Vila M., López-Herradón A., Ardura J.A., Mulero F., Gómez-Barrena E., Vallet-Regí M., Esbrit P. (2014). Parathyroid hormone-related protein (107–111) improves the bone regeneration potential of gelatin–glutaraldehyde biopolymer-coated hydroxyapatite. Acta Biomater..

[184] Ansari A.S., Santerre P.J., Uludağ H. (2017). Biomaterials for polynucleotide delivery to anchorage-independent cells. J. Mater. Chem. B.

[185] Gambari R., Brognara E., Spandidos D.A., Fabbri E. (2016). Targeting oncomiRNAs and mimicking tumor suppressor miRNAs: New trends in the development of miRNA therapeutic strategies in oncology (Review). Int. J. Oncol..

[186] Bonneau E., Neveu B., Kostantin E., Tsongalis G.J., De Guire V. (2019). How close are miRNAs from clinical practice? A perspective on the diagnostic and therapeutic market. Electron. J. Int. Fed. Clin. Chem. Lab. Med..

[187] Song G., Wang Q., Wang Y., Lv G., Li C., Zou R., Chen Z., Qin Z., Huo K., Hu R. (2013). A low-toxic multifunctional nanoplatform based on Cu9S5@mSiO2core-shell nanocomposites: Combining photothermal- and chemotherapies with infrared thermal imaging for cancer treatment. Adv. Funct. Mater..

[188] Salis A., Fanti M., Medda L., Nairi V., Cugia F., Piludu M., Sogos V., Monduzzi M. (2016). Mesoporous Silica Nanoparticles Functionalized with Hyaluronic Acid and Chitosan Biopolymers. Effect of Functionalization on Cell Internalization. ACS Biomater. Sci. Eng..

[189] Popat A., Liu J., Lu G.Q., Qiao S.Z. (2012). A pH-responsive drug delivery system based on chitosan coated mesoporous silica nanoparticles. J. Mater. Chem..

[190] Nairi V., Medda S., Piludu M., Casula M.F., Vallet-Regì M., Monduzzi M., Salis A. (2018). Interactions between bovine serum albumin and mesoporous silica nanoparticles functionalized with biopolymers. Chem. Eng. J..

[191] Xi J., Qin J., Fan L. (2012). Chondroitin sulfate functionalized mesostructured silica nanoparticles as biocompatible carriers for drug delivery. Int. J. Nanomed..

[192] Xu X., Lü S., Gao C., Bai X., Feng C., Gao N., Liu M. (2015). Multifunctional drug carriers comprised of mesoporous silica nanoparticles and polyamidoamine dendrimers based on layer-by-layer assembly. Mater. Des..

[193] Radhakrishnan K., Tripathy J., Datey A., Chakravortty D., Raichur A.M. (2015). Mesoporous silica—Chondroitin sulphate hybrid nanoparticles for targeted and bio-responsive drug delivery. N. J. Chem..

[194] Kavya K.C., Dixit R., Jayakumar R., Nair S.V., Chennazhi K.P. (2012). Synthesis and characterization of chitosan/chondroitin sulfate/nano- SiO_2_ composite scaffold for bone tissue engineering. J. Biomed. Nanotechnol..

[195] Porgham Daryasari M., Dusti Telgerd M., Hossein Karami M., Zandi-Karimi A., Akbarijavar H., Khoobi M., Seyedjafari E., Birhanu G., Khosravian P., SadatMahdavi F. (2019). Poly-l-lactic acid scaffold incorporated chitosan-coated mesoporous silica nanoparticles as pH-sensitive composite for enhanced osteogenic differentiation of human adipose tissue stem cells by dexamethasone delivery. Artif. Cells Nanomed. Biotechnol..

[196] Cao J.-F., Xu W., Zhang Y.-Y., Shu Y., Wang J.-H. (2020). Chondroitin sulfate-functionalized 3D hierarchical flower-type mesoporous silica with a superior capacity for selective isolation of low density lipoprotein. Anal. Chim. Acta.

[197] Argyo C., Cauda V., Engelke H., Rädler J., Bein G., Bein T. (2012). Heparin-coated colloidal mesoporous silica nanoparticles efficiently bind to antithrombin as an anticoagulant drug-delivery system. Chem. A Eur. J..

[198] Dai L., Li J., Zhang B., Liu J., Luo Z., Cai K. (2014). Redox-responsive nanocarrier based on heparin end-capped mesoporous silica nanoparticles for targeted tumor therapy in vitro and in vivo. Langmuir.

[199] Wan M.M., Yang J.Y., Qiu Y., Zhou Y., Guan C.X., Hou Q., Lin W.G., Zhu J.H. (2012). Sustained release of heparin on enlarged-pore and functionalized MCM-41. ACS Appl. Mater. Interfaces.

[200] Qian W.J., Wan M.M., Lin W.G., Zhu J.H. (2014). Fabricating a sustained releaser of heparin using SBA-15 mesoporous silica. J. Mater. Chem. B.

[201] Wei H., Han L., Ren J., Jia L. (2013). Anticoagulant surface coating using composite polysaccharides with embedded heparin-releasing mesoporous silica. ACS Appl. Mater. Interfaces.

[202] Wu F., Xu T., Zhao G., Meng S., Wan M., Chi B., Mao C., Shen J. (2017). Mesoporous Silica Nanoparticles-Encapsulated Agarose and Heparin as Anticoagulant and Resisting Bacterial Adhesion Coating for Biomedical Silicone. Langmuir.

